# Antagonism of prostate *α*_1A_-adrenoceptors by verapamil in human prostate smooth muscle contraction

**DOI:** 10.1016/j.jpet.2025.103603

**Published:** 2025-05-08

**Authors:** Sheng Hu, Guangyang Liu, Oluwafemi Kale, Wenbin Zhu, Yajie Xu, Patrick Keller, Philipp Weinhold, Alexander Tamalunas, Christian G. Stief, Martin Hennenberg

**Affiliations:** Department of Urology, LMU University Hospital, LMU Munich, Munich, Germany

**Keywords:** Benign prostatic hyperplasia, Lower urinary tract symptoms, Voiding symptoms, Prostate smooth muscle, *α*_1_-blocker, Verapamil

## Abstract

Voiding symptoms and hypertension are common comorbidities. *α*_1_-Blockers are the first-line medication for the treatment of voiding symptoms. Off-target antagonism of *α*_1_-adrenoceptors by cardiovascular drugs may add to the side effects of *α*_1_-blockers but may also hold the potential to avoid polypharmacy. Here, we examined *α*_1_-adrenergic antagonism by the calcium channel blocker verapamil in the human prostate. Prostate tissues were obtained from radical prostatectomy. Contractions were examined by organ bath. Verapamil caused concentration-dependent inhibitions of *α*_1_-adrenergic and electric field stimulation-induced contractions and increases of EC_50_ values for *α*_1_-agonists. E_max_ values for phenylephrine, methoxamine, noradrenaline, and electric field stimulation were decreased by 41%, 17%, 41%, and 39% by 1-*μ*M verapamil and by 62%, 36%, 51%, and 93% by 10-*μ*M verapamil. EC_50_ values for phenylephrine, methoxamine, and noradrenaline were increased by 0.47, 0.36, and 0.18 orders of magnitude by 1-*μ*M verapamil and by 0.83, 1.22, and 1.54 orders of magnitude by 10-*μ*M verapamil. The 100-nM verapamil increased the EC_50_ values for noradrenaline by 0.43 magnitudes but only slightly (<0.2 magnitudes) for phenylephrine and methoxamine. U46619-induced contractions were unchanged by 10-*μ*M verapamil. E_max_ values for endothelin-1-induced contractions were reduced by 14% by 10-*μ*M verapamil. Antagonism of *α*_1_-adrenoceptors by verapamil in the human prostate begins at concentrations corresponding to plasma concentrations at high doses. Improvements of voiding symptoms through this antagonism may help to avoid polypharmacy in elderly populations, but application in BPH may be limited by drug-drug interactions and additive side effects.

**Significance Statement:**

Our findings align verapamil concentrations antagonizing *α*_1_-adrenergic contractions of human prostate tissues with known plasma levels. Improvements of voiding symptoms appear possible, but application in benign prostatic hyperplasia may be limited by drug-drug interactions and additive side effects.

## 1 Introduction

Voiding symptoms suggestive of benign prostatic hyperplasia (BPH) and hypertension belong to the most common chronic diseases in men and show age-dependent prevalence (Lepor, 2004). About 50% of men aged 50–59 years, and 90% in the ninth decade show histologically proven BPH (Lepor, 2004), paralleled by micturition problems in 15% of men in the sixth, and up to 40% in the eighth life decade (Lepor, 2004). In turn, hypertension has been reported in 37% of men aged 40–59 years, 59% of men older than 59 years (Fryar et al, 2017), and 63% of men older than 65 years (McDonald et al, 2009). Thus, both conditions are highly prevalent comorbidities in the elderly, where multidrug regimens are a common problem, which will further increase with demographic transitions (Moßhammer et al, 2016; Page et al, 2019). Exemplarily, 42% of persons older than 65 years in Germany take at least 5 prescribed drugs, with nonprescribed medications not being included (Moßhammer et al, 2016). Problems arising from polypharmacy include the addition of side effects, poor adherence, and hospital admissions, among others (Gellad et al, 2011; Moßhammer et al, 2016).

The first-line option for medical treatment of voiding symptoms suggestive of BPH is *α*_1_-adrenoceptor antagonists (“*α*_1_-blockers”) (Gravas et al, 2023; Hennenberg and Michel, 2024). Side effects can be cardiovascular, resulting from inhibition of vasocontraction, and include orthostatic hypotension and dizziness (Gravas et al, 2023; Hennenberg and Michel, 2024). Side effects from *α*_1_-blockers may be additive with adverse events from other medications in multidrug regimens (Michel et al, 2001; Barendrecht et al, 2005; Hiremath et al, 2019). In fact, the incidence of *α*_1_-blocker-induced hypotension in the treatment of voiding symptoms is highest in patients with cardiovascular comorbidity and/or cardiovascular comedication (Barendrecht et al, 2005; Oelke et al, 2013). Even with tamsulosin, an *α*_1_-blocker with optimized subtype selectivity and only minor cardiovascular side effects in monotherapy, the risk for adverse events increases with the comedication of other *α*_1_-blocking agents, including the calcium channel blocker verapamil (Michel et al, 2001). In the elderly, where polypharmacy is common and who are most affected by BPH, side effects of *α*_1_-blockers in the treatment of voiding symptoms or hypertension may be associated with a slight, but significant risk of falls and fractures (Hiremath et al, 2019). Consequently, ongoing demographic transitions together with the age-dependent prevalence of chronic diseases call for strategies to reduce polypharmacy, in patients with BPH but also in general.

While additive side effects should be avoided, *α*_1_-adrenergic off-target antagonism of cardiovascular drugs could theoretically have the potential for the simultaneous treatment of voiding symptoms and hypertension (Hu et al, 2024a). Smooth muscle contraction in the hyperplastic prostate is a suspected, central factor in etiology of voiding symptoms and is induced by activation of *α*_1A_-adrenoceptors (Lepor, 2004; Hennenberg and Michel, 2024). Thus, voiding symptoms in BPH are commonly explained by urethral obstruction, driven by increased prostate smooth muscle tone and prostate enlargement (Lepor, 2004; Hennenberg and Michel, 2024). Symptom improvements by *α*_1_-blockers are believed to result from inhibition of *α*_1_-adrenergic contractions in the prostate, whereas antagonism of vascular *α*_1_-adrenoceptors accounts for cardiovascular side effects (Gravas et al, 2023; Hennenberg and Michel, 2024). The calcium channel blocker (calcium “antagonist”) verapamil shows the off-target antagonism of *α*_1_-adrenoceptors (Michel et al, 2001). Additive side effects with *α*_1_-blockers may contraindicate their comedication with *α*_1_-adrenergic antagonists (Michel et al, 2001) but also point to a potential for simultaneous benefits on cardiovascular diseases and voiding symptoms. However, its potential to replace *α*_1_-blockers in BPH treatment, in order to reduce polypharmacy, has never been considered, and the detailed preclinical data for off-target antagonism of *α*_1_-adrenergic contractions in the human prostate are not yet available. Here, we examined concentration-dependent effects of verapamil on contractions of human prostate tissues.

## 2 Materials and methods

### 2.1 Human prostate tissues

Human prostate tissues were obtained from radical prostatectomy for prostate cancer. This study was carried out in accordance with the Declaration of Helsinki of the World Medical Association and has been approved by the ethics committee of the Ludwig-Maximilians University, Munich, Germany. Informed consent was obtained from all participants. All procedures were performed in compliance with relevant laws and institutional guidelines and have been approved by the ethics committee at the faculty of medicine of the Ludwig-Maximilians University, Munich, Germany (approval number 22-0827, from 10-22-2022). The privacy rights of human subjects have been observed and informed consent was obtained from participants. Each sample was collected and examined anonymized. Typically, hormone or chemotherapy is only used in palliative situations or cases of metastatic prostate cancer. Accordingly, the patients in our study population were not treated with either hormone or chemotherapy prior to prostatectomy. Thus, the inclusion criteria included ≥18 years of age, individuals assigned male at birth, and radical prostatectomy for localized prostate cancer (≤T2). Exclusion criteria included prior prostate surgery for lower urinary tract symptoms/BPH, salvage prostatectomy, locally advanced prostate cancer (T3–4), prior chemotherapy, prior radiotherapy, and prior hormone therapy. Prostates from patients with previous surgery for BPH were excluded because this results in complete ablation of the periurethral zone. Tissues were anonymized immediately after sampling. Patient information was available prior to radical prostatectomy, and thus, patient selection for eligibility to voluntarily participate in our study could be performed. Eligible patients were presented with an informed consent form, which was obtained prior to radical prostatectomy. Until the surgery, informed consent could be revoked, without further consequences for the patient. After sampling and consecutive anonymization, this or retrospective correlation of experimental with clinical data were no longer possible.

Approximately 30–60 minutes after removal of prostates during surgery, macroscopical inspection and sampling were performed by a pathologist. For storage and transport, organs and samples were placed in Custodiol solution (Köhler). For macroscopic inspection and sampling, the prostate was opened by a single longitudinal cut from the capsule to the urethra, and both intersections were checked macroscopically for any obvious tumor infiltration. If no such infiltration was observed in the periurethral zone, tissues were taken from the transitional periurethral zone. In fact, tumor infiltration in this region was rare (<1% of prostates) because most prostate tumors occur in the peripheral zone (Pradidarcheep et al, 2011; Adler et al, 2012). Organ bath experiments were started within 3 h after sampling. The total number of experiments carried out was 95, whereas the estimated number of involved patients ranged around 80 but cannot be calculated in detail due to anonymization directly after sampling. The amounts of tissue sampled per prostate varied: some sampled tissue was only sufficient for 1 experiment with 3 channels (as described next), but in other cases, sampled tissues allowed 2 independent experiments (each with 4 channels).

### 2.2 Organ bath

Prostate tissues (3 × 3 × 6 mm) were mounted in organ baths (model 720 M, Danish Myotechnology), containing 4 chambers per device, stretched to stable pretensions (4.9 mN), and assessed for potassium chloride (KCl)-induced contractions (final concentration: 80 mM) for later reference of agonist- and electric field stimulation (EFS)-induced contractions, as described previously (Hu et al, 2024b). Cumulative concentration-response curves for agonists or frequency-response curves for EFS were constructed 30 minutes after addition of verapamil or solvent (Supplemental Figs. 1–18).

Each independent experiment included a verapamil and a control group, with tissues in both groups being obtained from the same organ. Only 1 concentration-response or frequency-response curve was recorded with each sample. Wherever possible, double determinations were performed. For double determinations, 2 organ bath channels were examined with verapamil, and the 2 others in the same device served as controls, using deionized water as the solvent for verapamil. From a total of 95 experiments, double determinations in both groups were possible in 81 experiments. In the remaining experiments, the amount of sampled tissues did not allow filling of 2 channels for both groups or samples did not contract with KCl so that single determinations were performed in 1 group or rarely (2 experiments) in both groups. However, each experiment included at least 1 sample for both groups, resulting in paired samples. Allocations of chambers to groups were changed between experiments.

Agonist- and EFS-induced contractions are expressed as percentage of 80 mM KCl-induced contractions, and E_max_ values, EC_50_ values for agonists, and frequencies (f) inducing 50% of maximum EFS-induced contraction (EF_50_) were calculated separately for each single experiment by curve fitting as previously reported (Huang et al, 2022; Hu et al, 2024b), using GraphPad Prism 6 (GraphPad Software Inc). Error messages are sent by the program if results from curve fitting are not plausible or if curve fitting failed (“ambiguous” and “interrupted”) occurred in 3 experiments (phenylephrine control group for 1 *μ*M verapamil, noradrenaline control group for 10 *μ*M verapamil, and U46619 control group). These curves contained downhill parts at high agonist concentrations, which were excluded and analyzed again, as recommended in the “GraphPad Curve Fitting Guide” (GraphPad Software, Inc), leading to plausible values.

To estimate the affinity of verapamil for *α*_1_-adrenoceptors in our experiments, “apparent” p*A*_2_ values were calculated as the sum of the negative decadic logarithm of the verapamil concentration and the right shift in concentration-response curves for *α*_1_-adrenoceptors, expressed as negative decadic logarithm: apparent p*A*_2_ = p(verapamil) + (pEC_50_ *α*_1_-agonist controls – pEC_50_ *α*_1_-agonist with verapamil). Values were calculated separately for each single experiment. The structure of our data differs from that required to calculate true p*A*_2_ values. The calculation of true p*A*_2_ values requires determinations with several ligand concentrations and a single, shared control group within the same experiment. However, our experimental design was based on 2 groups per experiment, involving 1 verapamil group and a separate control group for each verapamil concentration. The interpretation of apparent p*A*_2_ values may be subject to limitations and represents only an approximation to a true p*A*_2_ value or an estimate of affinity.

### 2.3 Statistical analyses

Data in concentration and frequency-response curves are means ± SD. E_max_, EC_50_, EF_50_, and p*A*_2_ values are presented as single values (means from double determination, where this was possible) together with means from all experiments in scatter plots. The calculation of MDs and 95% CIs (reported in the text), statistical analyses, and normality tests were performed using GraphPad Prism 6. The comparison of whole curves was performed by two-way ANOVA, without multiple comparison, as previously described (Huang et al, 2022). Normality within curves was assessed by testing the residuals of nonlinear regression fits using the Shapiro-Wilk test, applied separately to each control and verapamil group. The structure of the dataset, involving 3 variables and partly with repeated measurements, generated a sufficient number of residuals to permit normality testing despite group sizes of *n* = 5–7. Although test results suggested deviations from normal distribution in most cases (see Supplemental Table 1), two-way ANOVA was used due to the absence of nonparametric alternatives and its robustness against moderate violations of normality in small, paired samples. In contrast, our datasets containing E_max_, EC_50_, and EF_50_ values (*n* = 5–7 per group) were underpowered for valid application of recommended normality tests (D'Agostino and Pearson omnibus normality test and Shapiro-Wilk normality test). These tests are not supported in GraphPad Prism for our group sizes. Technically, a distribution analysis was still possible using the “Kolmogorov-Smirnov test with Dallal-Wilkinson–Lillie for *P* value” in GraphPad Prism, even though this approach is explicitly discouraged by the software for our data and is not considered valid. However, to obtain a rough, nonbinding indication of the data distribution, E_max_, EC_50_, and EF_50_ values were analyzed using the Kolmogoro-Smirnov test and visually inspected in parallel (see Supplemental Table 2). If a normal distribution could be reasonably assumed based on both test results and visual inspection, groups were compared by a paired Student’s *t* test. If nonnormality could confidently be assumed in at least 1 group, data were compared by the Wilcoxon matched-pairs signed rank test (applied to EC_50_ values for noradrenaline with 100 nM verapamil and controls, to EC_50_ values for endothelin-1, and to EF_50_ values with 100 nM, 1 *µ*M and 10 *µ*M verapamil and controls). If a *P* < .05 in the Kolmogorov-Smirnov test was not accompanied by clear visual deviations and the Wilcoxon results appeared biologically implausible, results from the Student’s *t* test are reported in the figure, and Wilcoxon *P* values are noted in the legend (applied to EC_50_ values for methoxamine and E_max_ values for EFS with 1 *µ*M verapamil and controls). *P* values < .05 were considered significant. The present study and analyses show an exploratory design because typical features of a strictly hypothesis-testing study were lacking, including a clear preset study plan, blinding, or biometric calculation of group sizes (Michel et al, 2020). Consequently, *P* values reported here need to be considered as descriptive but not as hypothesis testing (Michel et al, 2020), which also aligns with the limitations associated to normality testing of our data. Interpretation and discussion of results were based on effect sizes and their possible relevance, instead of *P* values. Minimum group sizes were preplanned as *n* = 5 for each series, to allow calculation of descriptive *p* values. Thus, series were discontinued after 5 independent experiments if it was obvious that no effect could be expected or if *P* values were <.05 between both groups in frequency/concentration-response curves. If these initial results were inconclusive, ie, pointed to a possible drug effect but with *P* values >.05, series were continued and analyzed again. Specifically, increasing group sizes after 5 initial experiments was applied to the series addressing effects of 1 *μ*M verapamil on phenylephrine-induced contractions, which finally included 6 independent experiments, and addressing effects of 100 nM on methoxamine- and EFS-induced contractions, each including finally 7 independent experiments. This procedure was possible due to the explorative character, as long as it is reported in detail (Michel et al, 2020). Interim analyses were limited to frequency and concentration-response curves and did not include E_max_, EC_50_, and EF_50_ values, which were calculated only after completion of series. No data or experiments were excluded from analyses, apart from downhill parts in concentration-response curves during curve fitting of 3 experiments, as described earlier.

### 2.4 Drugs and nomenclature

Verapamil hydrochloride was obtained from Tocris. Stock solutions (1000-fold of final concentrations) were prepared with deionized water and stored as aliquots at −20 °C. Noradrenaline, phenylephrine, and methoxamine were obtained from Sigma-Aldrich. Stock solutions of phenylephrine, methoxamine, and noradrenaline with distilled water were freshly prepared before each experiment. U46619 is an agonist of the thromboxane A_2_ receptor and was dissolved in ethanol. Stock solutions (10 mM) were stored at −80 °C until use. Endothelin-1 was dissolved in DMSO and stock solutions (0.4 mM) were stored at −20 °C until use. U46619 and endothelin-1 were obtained from Enzo Life Sciences.

## 3 Results

### 3.1 Effects on phenylephrine-induced contractions

**Fig. 1:**
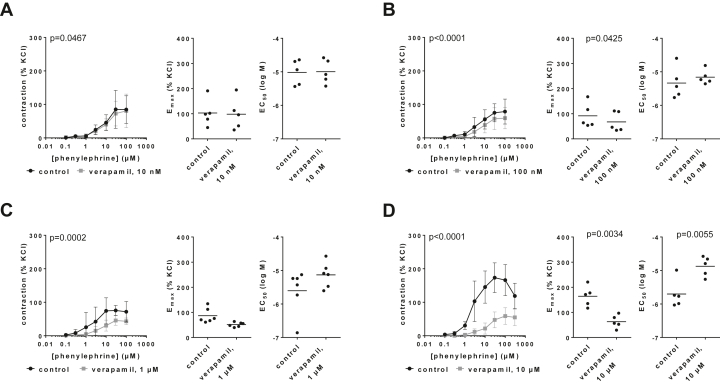
Effects of verapamil on phenylephrine-induced contractions of human prostate tissues. Contractions were induced by phenylephrine, 30 minutes after the addition of 10 nM (A), 100 nM (B), 1 *μ*M (C), or 10 *μ*M verapamil (D), or of an equivalent amount of deionized water to controls. Data are from 5 independent experiments (A, B, D) and 6 independent experiments (C) per diagram, where tissues from 5 (A, B, D) or 6 patients (C) were split to both groups of a panel (ie, verapamil and control), resulting in paired samples. Data are shown as means ± SD from all experiments in concentration-response curves together with *P* values from two-way ANOVA, and all single E_max_ and EC_50_ values from each experiment (calculated by curve fitting) in scatter plots together with *P* values from paired Student’s *t* tests.

Verapamil caused concentration-dependent inhibitions of phenylephrine-induced contractions of human prostate tissues and concentration-dependent increases in EC_50_ values for phenylephrine (Fig. 1; Supplemental Figs. 1–4). Contractions and EC_50_ values for phenylephrine were obviously unchanged with 10-nM verapamil (Fig. 1A). Contractions in concentration-response curves and E_max_ values for phenylephrine were concentration-dependently reduced by 100 nM, 1 *μ*M, and 10 *μ*M verapamil. E_max_ values decreased from 92% (31–153) of KCl-induced contractions in controls to 68% (19–116) with 100 nM (Fig. 1B), from 87% (56–118) in controls to 51% (42–61) with 1 *μ*M (Fig. 1C), and from 164% (114–214) to 63% (31–95) with 10 *μ*M verapamil (Fig. 1D). EC_50_ values for phenylephrine (log M) mounted to −5.34 (−5.91 to −4.76) in controls and −5.16 (−5.42 to −4.9) with 100-nM verapamil (Fig. 1B) and were increased from −5.6 (−6.29 to −4.92) in controls to −5.13 (−5.52 to −4.74) with 1 *μ*M (MD 0.47 [−0.32 to 1.27]) (Fig. 1C) and from −5.7 (−6.22 to −5.18) in controls to −4.88 (−5.24 to −4.51) with 10 *μ*M verapamil (MD 0.83 [0.41–1.25]) (Fig. 1D).

### 3.2 Effects on methoxamine-induced contractions

**Fig. 2:**
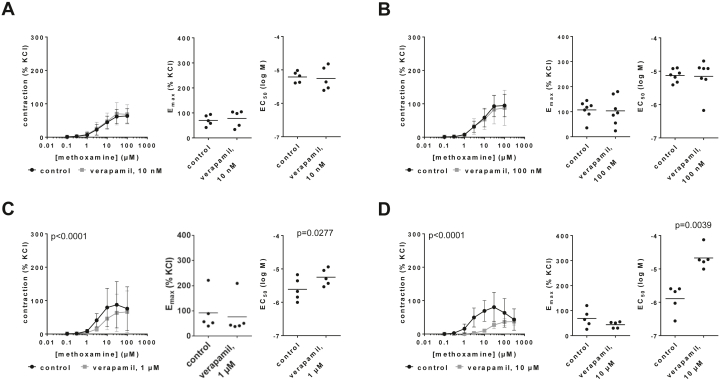
Effects of verapamil on methoxamine-induced contractions of human prostate tissues. Contractions were induced by methoxamine, 30 minutes after the addition of 10 nM (A), 100 nM (B), 1 *μ*M (C), or 10 *μ*M verapamil (D), or of an equivalent amount of deionized water to controls. Data are from 5 independent experiments (A, C, D) and 7 independent experiments (B) per diagram, where tissues from 5 (A, C, D) or 7 patients (B) were split to both groups of a panel (ie, verapamil and control), resulting in paired samples. Data are shown as means ± SD from all experiments in concentration-response curves together with *P* values from two-way ANOVA, and all single E_max_ and EC_50_ values from each experiment (calculated by curve fitting) in scatter plots together with *P* values from paired Student’s *t* tests. Because deviations from normal distribution could be tentatively assumed for E_max_ values in (C) and EC_50_ values in (D), these data were additionally analyzed using the Wilcoxon matched-pairs signed rank test, which suggested no significant differences (*P* = .0625 in both datasets).

Verapamil caused concentration-dependent inhibitions of methoxamine-induced contractions of human prostate tissues and concentration-dependent increases in EC_50_ values for methoxamine (Fig. 2; Supplemental Figs. 5–8). Contractions and EC_50_ values for methoxamine were obviously unchanged with 10 nM (Fig. 2A) and with 100-nM verapamil (Fig. 2B). Concentration-response curves were right shifted by 1-*μ*M (Fig. 2C) and 10-*μ*M verapamil (Fig. 2D), including inhibition at submaximal methoxamine concentrations, full (Fig. 2C) or partial (Fig. 2D) recovery at high methoxamine concentrations, and unchanged or only slightly reduced E_max_ values. E_max_ values mounted to 92% (−1 to 185) of KCl-induced contractions in controls and 76% (−16 to 169) with 1 *μ*M verapamil (Fig. 2C) and to 69% (24–114) in controls and 44% (28–59) with 10 *μ*M verapamil (Fig. 2D). EC_50_ values for methoxamine (log M) mounted to −5.13 (−5.3 to −4.95) in controls and −5.15 (−5.61 to −4.7) with 100 nM verapamil (Fig. 2B) and were increased from −5.61 (−6.04 to −5.19) in controls to −5.24 (−5.56 to −4.94) with 1 *μ*M (MD 0.36 [0.06–0.66]) (Fig. 2C) and from −5.89 (−6.41 to −5.38) in controls to −4.67 (−5.08 to −4.27) with 10 *μ*M verapamil (MD 1.22 [0.65–1.78]) (Fig. 2D).

### 3.3 Effects on noradrenaline-induced contractions

**Fig. 3:**
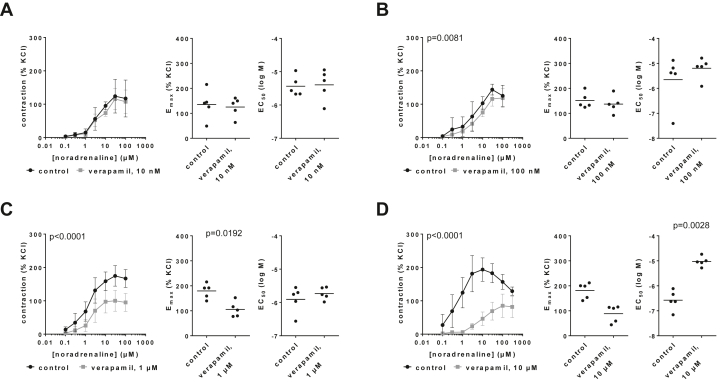
Effects of verapamil on noradrenaline-induced contractions of human prostate tissues. Contractions were induced by noradrenaline, 30 minutes after the addition of 10 nM (A), 100 nM (B), 1 *μ*M (C), or 10 *μ*M verapamil (D), or of an equivalent amount of deionized water to controls. Data are from 5 independent experiments per diagram, where tissues from 5 patients were split into both groups of a panel (ie, verapamil and control), resulting in paired samples. Data are shown as means ± SD from all experiments in concentration-response curves together with p values from two-way ANOVA, and all single E_max_ and EC_50_ values from each experiment (calculated by curve fitting) in scatter plots together with p values from paired Student’s *t* tests.

Verapamil caused concentration-dependent inhibitions of noradrenaline-induced contractions of human prostate tissues and increases in EC_50_ values for noradrenaline (Fig. 3; Supplemental Figs. 9–12). Contractions and EC_50_ values for noradrenaline were obviously unchanged with 10 nM (Fig. 3A). Contractions in concentration-response curves were slightly reduced by 100-nM verapamil (Fig. 3B) and obviously reduced by 1 and 10 *μ*M verapamil. E_max_ values mounted to 151% (112–190) of KCl-induced contractions in controls and 137% (93–180) with 100 nM verapamil (Fig. 3B) and decreased from 179% (142–217) in controls to 106% (68–144) with 1 *μ*M verapamil (Fig. 3C) and from 181% (142–220) in controls to 88% (46–131) with 10 *μ*M verapamil (Fig. 3D). Concentration-response curves for noradrenaline were obviously right shifted by 10 *μ*M verapamil (Fig. 3D). EC_50_ values for noradrenaline (log M) mounted to −5.65 (−6.897 to −4.41) in controls and −5.196 (−5.73 to −4.67) with 100-nM verapamil (MD 0.46 [−1.08 to 1.995]) (Fig. 3B) and −5.91 (−6.4 to −5.42) in controls and −5.73 (−5.96 to −5.5) with 1 *μ*M verapamil (MD 0.18 [−0.24 to 0.6]) (Fig. 3C) and increased from −6.58 (−7.06 to −6.09) in controls to −5.03 (−5.27 to −4.78) with 10 *μ*M verapamil (MD 1.54 [0.9–2.2]) (Fig. 3D).

### 3.4 Apparent pA_2_ values

**Fig. 4:**
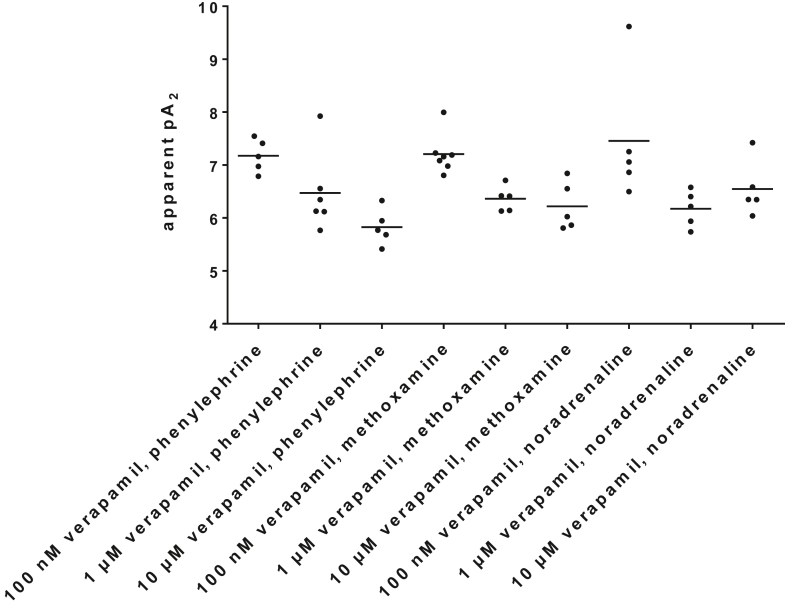
Apparent p*A*_2_ values of verapamil. Apparent p*A*_2_ values were calculated for series with 100 nM, 1 *μ*M, and 10 *μ*M verapamil, applied to phenylephrine, methoxamine, and noradrenaline. The calculation was based on experiments shown in Fig. 1, Fig. 2, Fig. 3. Shown are all values together with SD from all single, independent experiments.

Apparent p*A*_2_ values were calculated for series with 100 nM, 1 *μ*M and 10 *μ*M verapamil, applied to phenylephrine, methoxamine, and noradrenaline (Fig. 4), from experiments reported above (Fig. 1, Fig. 2, Fig. 3). The calculation was possible for all experiments in these series. The average apparent p*A*_2_ values of all 9 series ranged between 7.457 and 5.828 (corresponding to 37 nM–1.49 *μ*M) and were most consistent across the 6 series with 1- and 10-*μ*M verapamil (Fig. 4). Specifically, apparent p*A*_2_ values in experiments with phenylephrine, methoxamine, and noradrenaline mounted to 7.18 (6.79–7.56), 7.21 (6.86–7.56), and 7.46 (5.92–8.995) with 100 nM verapamil (corresponding to 67, 75, and 37 nM); to 6.47 (5.68–7.27), 6.36 (6.07–6.66), and 6.18 (5.75–6.6) with 1 *μ*M verapamil (corresponding to 347, 434, and 668 nM); and to 5.83 (5.41–6.25), 6.22 (5.65–6.78), and 6.548 (5.895–7.2) with 10 *μ*M verapamil (corresponding to 1.49 *μ*M, 604 nM, and 283 nM) (Fig. 4).

### 3.5 Effects on EFS-induced contractions

**Fig. 5:**
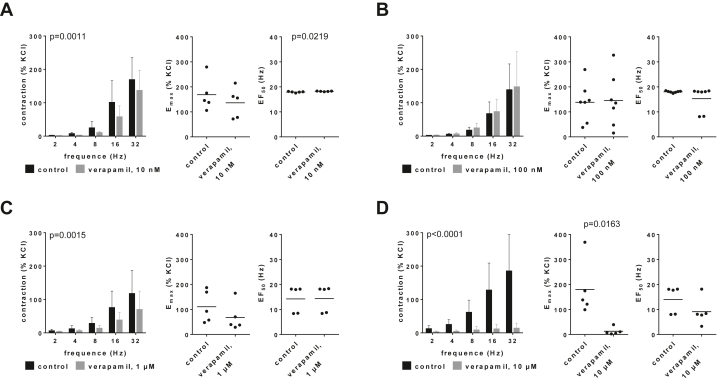
Effects of verapamil on EFS-induced contractions of human prostate tissues. Contractions were induced by EFS, 30 minutes after the addition of 10 nM (A), 100 nM (B), 1 *μ*M (C), or 10 *μ*M verapamil (D), or of an equivalent amount of deionized water to controls. Data are from 5 independent experiments (A, C, D) and 7 independent experiments (B) per diagram, where tissues from 5 (A, C, D) or 7 patients (B) were split to both groups of a panel (ie, verapamil and control), resulting in paired samples. Data are shown as means ± SD from all experiments in frequency-response curves together with *P* values from two-way ANOVA, and all single E_max_ and EF_50_ values from each experiment (calculated by curve fitting) in scatter plots together with *P* values from paired Student’s *t* tests. Because deviations from normal distribution could be tentatively assumed for E_max_ values in (D) and EF_50_ values in (B)–(D), these data were additionally analyzed using the Wilcoxon matched-pairs signed rank test, which suggested no significant differences (*P* = .0625 for E_max_ values in [D]).

Verapamil caused inhibitions of EFS-induced contractions of human prostate tissues, which occurred using concentrations of 10 nM, 1 *μ*M, and 10 *μ*M and were largest with 10 *μ*M (Fig. 5; Supplemental Figs. 13–16). E_max_ values for EFS-induced inhibitions were reduced from 169% (86–251) of KCl-induced contractions in controls to 136% (61–212) with 10 nM verapamil (Fig. 5A), mounted to 138% (66–210) in controls and 145% (47–243) with 100 nM (Fig. 4B), and were reduced from 111% (31–191) in controls to 68% (−2 to 138) with 1 *μ*M (Fig. 5C) and from 180% (45–316) in controls to 13% (−6 to 32) with 10 *μ*M verapamil (Fig. 5D). A possible effect on EF_50_ occurred with 10 *μ*M, mounting to 14 Hz (7–21) in controls and 9 Hz (2–16) with verapamil (Fig. 5D).

### 3.6 Effects on nonadrenergic contractions

**Fig. 6:**
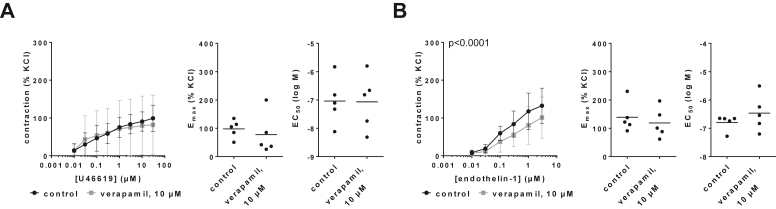
Effects of verapamil on U46619- and endothelin-1-induced contractions of human prostate tissues. Contractions were induced by U46619 (A) or endothelin-1 (B), 30 minutes after the addition of 10 *μ*M verapamil or an equivalent amount of deionized water to controls. Data are from 5 independent experiments per diagram, where tissues from 5 patients were split into both groups of a panel (ie, verapamil and control), resulting in paired samples. Data are shown as means ± SD from all experiments in concentration-response curves together with *P* values from two-way ANOVA, and all single E_max_ and EC_50_ values from each experiment (calculated by curve fitting) in scatter plots together with *P* values from paired Student’s *t* tests.

Verapamil (10 *μ*M) had no effects on U46619-induced contractions of human prostate tissues, reflected by unchanged concentration-response curves, E_max_ values and EC_50_ values (Fig. 6A; Supplemental Fig. 17). Concentration-response curves for endothelin-1-induced contractions were slightly reduced by 10 *μ*M verapamil (Fig. 6B; Supplemental Fig. 18), reflected by E_max_ values for endothelin-1-induced contractions of 139% (72–206) in controls and 119% (53–185) with verapamil (Fig. 6B). EC_50_ values for endothelin-1 were not changed by verapamil (Fig. 6B).

## 4 Discussion

Verapamil is an antagonist of voltage-gated calcium channels (CaV), inhibiting CaV1.2 with IC_50_ values from 0.3 to 5 *μ*M, and CaV1.1 with IC_50_ values around 10 *μ*M (Alexander et al, 2021), and is used for the treatment of cardiovascular diseases. As an off-target effect, it antagonizes *α*_1_-adrenoceptors, which has been previously reported from competition assays with (^3^H)-prazosin (Nishimura et al, 1986; Thoolen et al, 1987; Müller and Noack, 1988). The clinically applied drug represents a racemate, containing a D- and L-enantiomer. Affinities for *α*_1_-adrenergic receptors may differ with subtype, model, and enantiomers. Verapamil (probably the racemate, if not other stated) replaced (^3^H)-prazosin with an affinity of 660 nM in membrane preparations from porcine aortae (Nishimura et al, 1986), 620 nM for the racemate, 280 nM for the L-enantiomer, and 1.67 *μ*M for the D-enantiomer in membrane preparations from guinea pig cardiac myocytes (Müller and Noack, 1988), 600 nM in rat heart membranes (Karliner et al, 1982), 600 nM in rat left ventricle, 2 *μ*M in rat renal cortex and 1 *μ*M in BC3H-1 cell membranes (Motulsky et al, 1983), 4.77 *μ*M for the (−) and 6.82 *μ*M for the (+) enantiomer in rat cardiac membranes (Nayler et al, 1982), and 9.7 *μ*M in cerebral membranes from rats (Thoolen et al, 1987). These previously reported affinities are fully in the ranges of our apparent p*A*_2_ values, mostly suggesting affinities of 283–668 nM, or up to 1.49 *μ*M in 1 of our 9 series of experiments with *α*_1_-agonists, addressing antagonism of *α*_1_-adrenoceptors in smooth muscle contraction of human prostate tissues. Probably, our values reflect the affinity to *α*_1A_, which is the predominant or single subtype involved in human prostate smooth muscle contraction (Hennenberg and Michel, 2024). Species- and tissue-dependent subtype composition of *α*_1_-adrenoceptor populations may generally affect binding of *α*_1_-adrenergic ligands in preclinical models. Approaches, including different concentrations of verapamil, have often been used to determine affinities in competition assays, but rarely in contraction experiments with tissues. To the best of our knowledge, this study is the first systematic investigation in human prostate tissues, which is based on a comprehensive approach.

*α*_1_-Adrenoceptor antagonists are the first-line option and gold standard in the medical treatment of voiding symptoms. Though their efficacy is limited, they reduce international prostate symptom scores by 30%–50% and enhance the maximum urinary flow rate up to 20%–40% in controlled studies with placebo run-in (Hennenberg and Michel, 2024). In fact, *α*_1_-blockers represent the most prescribed drug class for treatment of voiding symptoms suggestive of BPH (Tamalunas et al, 2024). Consequently, drugs showing antagonism of *α*_1_-adrenoceptors and inhibition of prostate smooth muscle contractions in preclinical models may be considered promising to improve voiding symptoms in BPH in vivo if it occurs with concentrations in the ranges of plasma levels. Using a single oral dose of 120-mg verapamil, maximum plasma levels mount to 220 nM, obtained after 1 h (Johnston et al, 1981). Chronic treatment is possible with doses of 240–480 mg/day, resulting in plasma levels of 880 nM with 4 × 120 mg/day (FDA, 2013) or 1.9 *μ*M with 2 daily, oral doses of 240 mg (Anderson et al, 1986). Thus, the p*A*_2_ values we estimated, and also most of the previously reported affinities for *α*_1_-adrenoceptors are within the range of possible plasma concentrations. Effects on contractions and EC_50_ values of *α*_1_-adrenergic contractions in our experiments were still limited, but obvious using 1 *μ*M verapamil, and became sustained with 10 *μ*M. However, IC_50_ values for CaV1.2 range between 0.3 and 5 *μ*M, which is obviously sufficient for cardiovascular effects in vivo. Together, the balance of concentrations to effect sizes in our experiments appears to be right on the border of possible in vivo effects and plasma concentrations. Based on our findings together with known plasma levels, the effects of verapamil on voiding symptoms in vivo may not be fully excluded. However, effects on voiding symptoms certainly require maximum dosing of up to 480 mg/day, and inhibitory effects as seen with 10 *μ*M in our study will not occur even with maximum available doses in vivo.

While *α*_1_-blockers are recommended and routinely used for treatment of voiding symptoms in BPH, their application may be limited by side effects (Gravas et al, 2023), in particular in elderly, who in turn are most affected by lower urinary tract symptoms (Lepor, 2004). First, side effects of *α*_1_-blockers may be additive with those of other medications, becoming most problematic in multidrug regimens, which are common in elderly patients (Bhanu et al, 2024). Second, the most frequent side effects of *α*_1_-blockers include orthostatic hypertension and dizziness (Gravas et al, 2023; Hennenberg and Michel, 2024), with an associated tendency to falls and fractures, for which the risk is already increased without *α*_1_-blockers in older patients (Bhanu et al, 2024; Jansen and van der Velde, 2024). Thus, new strategies to reduce polypharmacy in elderly patients may be highly desirable, in view of ongoing demographic transitions, of the prevalence and consequences of multidrug regimens (Bhanu et al, 2024), and the age-dependent prevalence of voiding symptoms, BPH, and hypertension (Lepor, 2004; McDonald et al, 2009; Fryar et al, 2017). Assuming that verapamil effectively reduces voiding symptoms in patients with BPH, it may be theoretically used to replace *α*_1_-blockers or other BPH-specific drugs, with simultaneous treatment of hypertension. Similar concepts have been recently suggested for the *β*-blocker carvedilol, again showing off-target antagonism of *α*_1_-adrenoceptors in human prostate smooth muscle contraction, potentially starting in ranges of plasma concentrations (Hu et al, 2024a).

However, a targeted use of verapamil in BPH is subject to limitations and dependent on further studies. Apart from vascular smooth muscle, verapamil acts on calcium channels in the myocardium, where it can cause bradycardia and negative inotropism, among other systemic effects (Fahie and Cassagnol, 2025). In fact, guidelines for treatment of hypertension recommend or prefer dihydropyridine calcium channel blockers, but not nondihydropyridines such as verapamil (Unger et al, 2020; McEvoy et al, 2024). Based on its cardiac effects, verapamil is meanwhile used for cardiovascular conditions other than hypertension, including supraventricular tachycardia or angina (Fahie and Cassagnol, 2025). Verapamil has a number of contraindications in elderly patients, who are most affected by BPH but who are also more prone to cardiovascular complications such as sinus disease, cardiomyopathy, renal impairment, bundle branch block, and others (Fahie and Cassagnol, 2025). In addition, pharmacokinetics can be highly erratic in elderly populations. Together, the application of verapamil at high doses (480 mg/day) in elderly patients could pose significant risks compared to the use of specific *α*_1_-blockers like tamsulosin.

Calcium channel blockers have been generally suspected of causing lower urinary tract symptom and urinary retention, but evidence is in fact poor and lacking for verapamil. Although drugs that inhibit contractions of both the detrusor and the prostate may cause urinary retention in BPH, combination therapies are also routinely used to treat voiding symptoms and overactive bladder simultaneously (Oelke et al, 2013). Using 1 *μ*M, verapamil completely inhibited potassium-induced and halfed carbachol-induced contractions of isolated human detrusor tissues (Fovaeus et al, 1987; Badawi et al, 2006). In patients with bladder hyperactivity, though not in patients with detrusor instability, intravesical instillation of solutions with 30 *μ*M verapamil increased the bladder capacity (Mattiasson et al, 1989). However, this concentration is out of physiological ranges, while lower concentrations have not been applied (Mattiasson et al, 1989). Male and female calcium channel blocker users showed higher international prostate symptom scores compared to nonusers (Elhebir et al, 2013). Similarly, the mean international prostate symptom score in male patients increased from 3.1 points before treatment initiation to 9.8 after commencing treatment with calcium channel blockers for cardiovascular diseases (Hughes et al, 2011). However, only 2 of 85 patients or 2 of 38 patients in the treatment groups were using verapamil (Hughes et al, 2011; Elhebir et al, 2013) so that specific conclusions are not possible. None of these 2 studies reported urinary retention under treatment with calcium channel blockers. Thus, verapamil-induced urinary retention may depend on patients’ conditions and does not occur in general but needs to be considered and assessed before targeted use in BPH.

Attempts to reduce adverse events in treatment of voiding symptoms with *α*_1_-blockers resulted in the introduction of tamsulosin and silodosin (Hennenberg and Michel, 2024). Their affinity has been optimized for *α*_1A_, whereas cardiovascular effects may involve *α*_1B_ and/or *α*_1D_ as well (Hennenberg and Michel, 2024). Available antagonists for treatment of voiding symptoms include subtype-unselective compounds (alfuzosin, doxazosin, and terazosin), tamsulosin with reduced affinity for *α*_1B_, and the highly *α*_1A_-selective silodosin (Gravas et al, 2023; Hennenberg and Michel, 2024). The prevalence of hypotension is in fact reduced with tamsulosin alone (Gravas et al, 2023; Hennenberg and Michel, 2024), but the incidence of adverse events (including cardiovascular) increases substantially by its unintentional or erroneous comedication with other *α*_1_-blocking agents, including verapamil or other *α*_1_-blockers (eg, if prescribed for reduction of blood pressure) (Michel et al, 2001). At least in elderly patients (>65 years), the risk of fall and fracture is increased with *α*_1_-blockers in general (Schimke and Schinike, 2014; Seo et al, 2015; Testa et al, 2018; McDonnell et al, 2020) and even slightly with tamsulosin and silodosin despite their optimized subtype selectivity (Welk et al, 2015). While a tendency to fall may not be relevant in younger patients, side effects from *α*_1_-blockers may contribute to high discontinuation rates in the treatment of voiding symptoms, finally resulting in progression and complications (Cindolo et al, 2015a,b).

The risk to experience orthostatic hypotension is cumulative in multidrug regimens (Bhanu et al, 2024). Commonly prescribed drugs potentially causing orthostatic hypotension are angiotensin-converting enzyme inhibitors, calcium channel blockers, *β*-blockers, selective serotonin reuptake inhibitors, and *α*_1_-blockers (Bhanu et al, 2024). In men aged ≥70 years, combinations of antihypertensives (*β*-blocker, angiotensin-converting enzyme inhibitor, and calcium channel blockers) with *α*_1_-blockers belong to the most commonly prescribed multidrug clusters (Bhanu et al, 2024). In men aged 50–69 years, the most commonly prescribed drug clusters are combinations of antihypertensives (angiotensin receptor blockers, *β*-blockers, angiotensin-converting enzyme inhibitors, and calcium channel blockers) with antidepressants (selective serotonin reuptake inhibitors and tricyclic antidepressants) (Bhanu et al, 2024). In view of demographic transitions and the increase in polypharmacy, improved knowledge on drug interactions with relevance for elderly patient groups and new strategies to reduce polypharmacy are in demand. Our current findings suggest that comedication of verapamil (eg, as an antihypertensive) with *α*_1_-blockers should be avoided, but that verapamil could even replace *α*_1_-blockers to reduce polypharmacy and may be used for simultaneous treatment of hypertension and voiding symptoms.

In rats with surgically induced, partial urethral obstruction, intravenously applied verapamil reduced voiding and increased the pressure needed to initiate voiding (Saito and Kondo, 1998). These findings rather reflect effects on bladder than prostate function or on storage than on voiding symptoms, possibly by inhibition of Cav1.2 in the detrusor. Cav1.2 inhibitions may have contributed to reductions in maximum contractions in our experiments as well, in addition to antagonism of *α*_1_-adrenoceptors. While shifts and increases in EC_50_ values for *α*_1_-agonists by verapamil were obvious and point to antagonism of *α*_1A_, parallel decreases in maximum contractions (reflected by decreases in E_max_ values) became evident as well, in particular in series using 10 *μ*M. The inhibition of the EFS-induced contractions was already strong with 1 *μ*M. At the latest with 10 *μ*M, which virtually fully inhibited EFS-induced contractions, effect sizes were reached that were previously observed with tamsulosin or silodosin under the same conditions (Wang et al, 2020; Hu et al, 2023). Again, effects of verapamil on EFS-induced contractions may be mixed, including components from CaV1.2 inhibition and antagonism of *α*_1A_-adrenoceptors. EFS-induced prostate smooth muscle contractions are caused by adrenergic neurotransmission and subsequent activation of postsynaptic *α*_1_-adrenoceptors on smooth muscle cells. In addition to *α*_1A_-adrenoceptors, prostate smooth muscle contraction can be induced by activation of thromboxane A_2_ and endothelin receptors, which are supposed to contribute to urethral obstruction and thus to medication-refractory voiding symptoms. Our concentration-response curves for U46619 and endothelin-1 provided no signs for antagonism of these receptors by verapamil. A small inhibition of endothelin-1-induced contractions by 10 *μ*M verapamil was probably attributed to CaV1.2 inhibition, but not seen in experiments with U46619, suggesting that intracellular mechanisms in thromboxane- and endothelin-induced contractions are not fully the same. In fact, receptor-, organ-, and species-dependent differences in agonist-induced smooth muscle contractions have been exemplarily documented (Tamalunas et al, 2022) but are still poorly understood.

The affinity of verapamil is probably highest for calcium channels (CaV1.1). In contrast to its off-target effects at *α*_1_-adrenoceptors, only a few studies allowed conclusions regarding possible binding to *α*_2_-adrenoceptors. Two studies suggested strong inhibitions, ie, strong decreases in E_max_ of *α*_2_-adrenoceptor-agonist-induced contractions of rat aortic rings by 10-*μ*M verapamil. With 10-*μ*M verapamil, contractions by the *α*_2B_-adrenergic agonist dexmedetomidine did not recover within the applied range (1 nM–1 *μ*M) (Ok et al, 2011) so that a possible right shift or antagonism cannot be fully excluded. Using subtype-unselective *α*_2_-adrenergic agonist UK14,304, a possible right shift could be estimated to be somewhat more than half a magnitude (Suenaga and Kamata, 2000). Considering that the affinity is apparently lower than for *α*_1_-adrenoceptors and that right shifts may be limited to 10 *μ*M, the relevance of binding to *α*_2_-adrenoceptors in clinical assessments and contributions to our findings may be limited.

## Conflict of interest

The authors declare no conflicts of interest.
