# Supplementary material for: Antagonism of prostate α1A-adrenoceptors by verapamil in human prostate smooth muscle contraction
Source: J Pharmacol Exp Ther. 2025 May 8;392(7):103603. doi: 10.1016/j.jpet.2025.103603 (PMC12405930; doi:10.1016/j.jpet.2025.103603)
Supplement: Supplementary Figures 1-18 [file mmc1.pdf]

Antagonism of prostate α1A-adrenoceptors by verapamil in human prostate smooth muscle contraction

Sheng Hu, Guangyang Liu, Oluwafemi Kale, Wenbin Zhu, Yajie Xu, Patrick Keller, Philipp Weinhold, Alexander Tamalunas, Christian G. Stief, Martin Hennenberg

Journal of Pharmacology and Experimental Therapeutics
JPET-D-25-00098R1

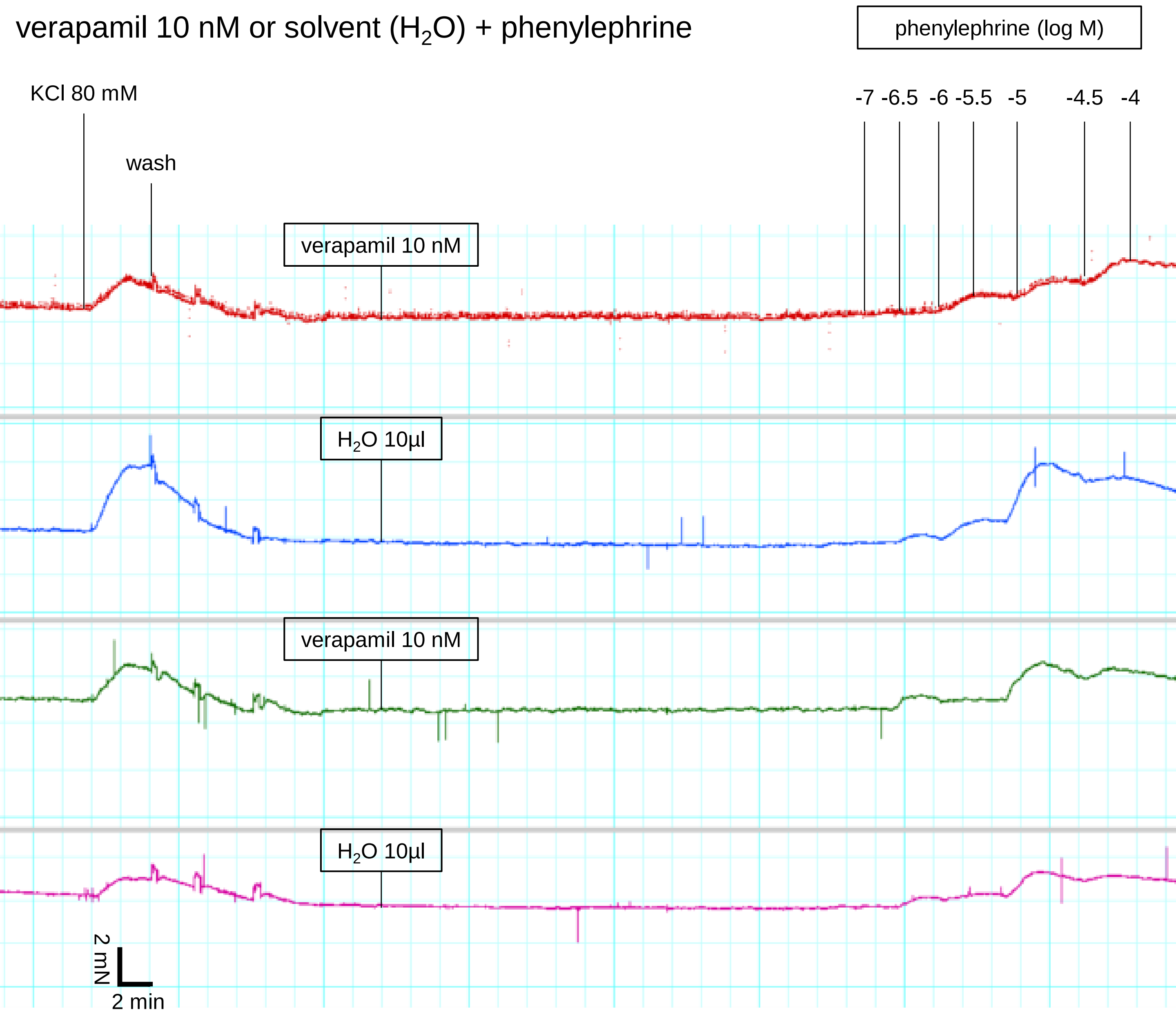

Supplemental Figure 1: Original tracings from an experiment adressing effects of 10 nM verapamil or solvent (control for verapamil) on concentration response curves for phenylephrine. All four curves were recorded with tissues from the same prostate and within the same experiment, which belongs to a series of n=5 independent experiments performed with tissues from n=5 patients (fig. 1a).

Antagonism of prostate α$_{1A}$-adrenoceptors by verapamil in human prostate smooth muscle contraction

Sheng Hu, Guangyang Liu, Oluwafemi Kale, Wenbin Zhu, Yajie Xu, Patrick Keller, Philipp Weinhold, Alexander Tamalunas, Christian G. Stief, Martin Hennenberg

Journal of Pharmacology and Experimental Therapeutics
JPET-D-25-00098R1

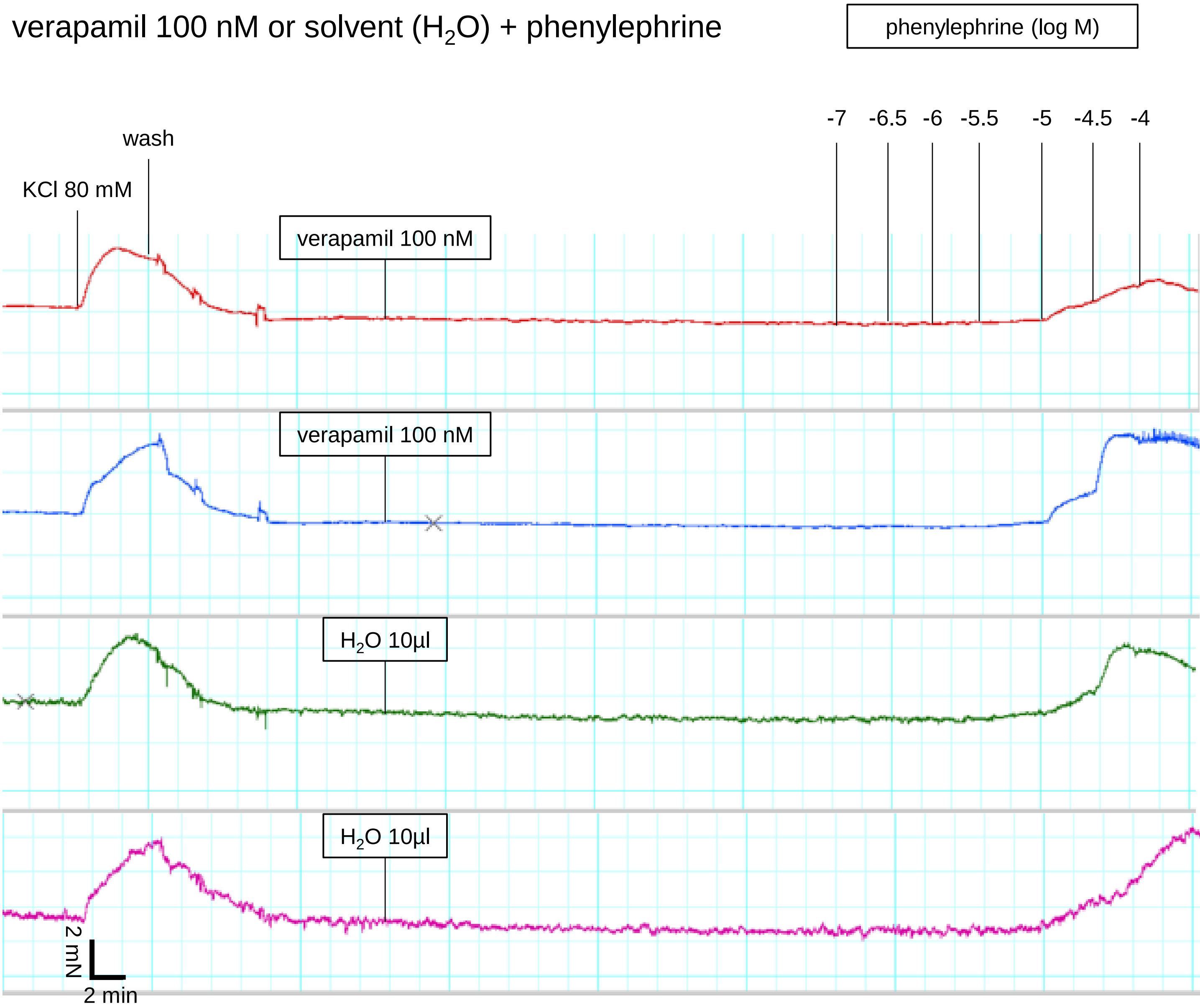

Supplemental Figure 2: Original tracings from an experiment adressing effects of 100 nM verapamil or solvent (control for verapamil) on concentration response curves for phenylephrine. All four curves were recorded with tissues from the same prostate and within the same experiment, which belongs to a series of n=5 independent experiments performed with tissues from n=5 patients (fig. 1b).

Sheng Hu, Guangyang Liu, Oluwafemi Kale, Wenbin Zhu, Yajie Xu, Patrick Keller, Philipp Weinhold, Alexander Tamalunas, Christian G. Stief, Martin Hennenberg
Journal of Pharmacology and Experimental Therapeutics
JPET-D-25-00098R1

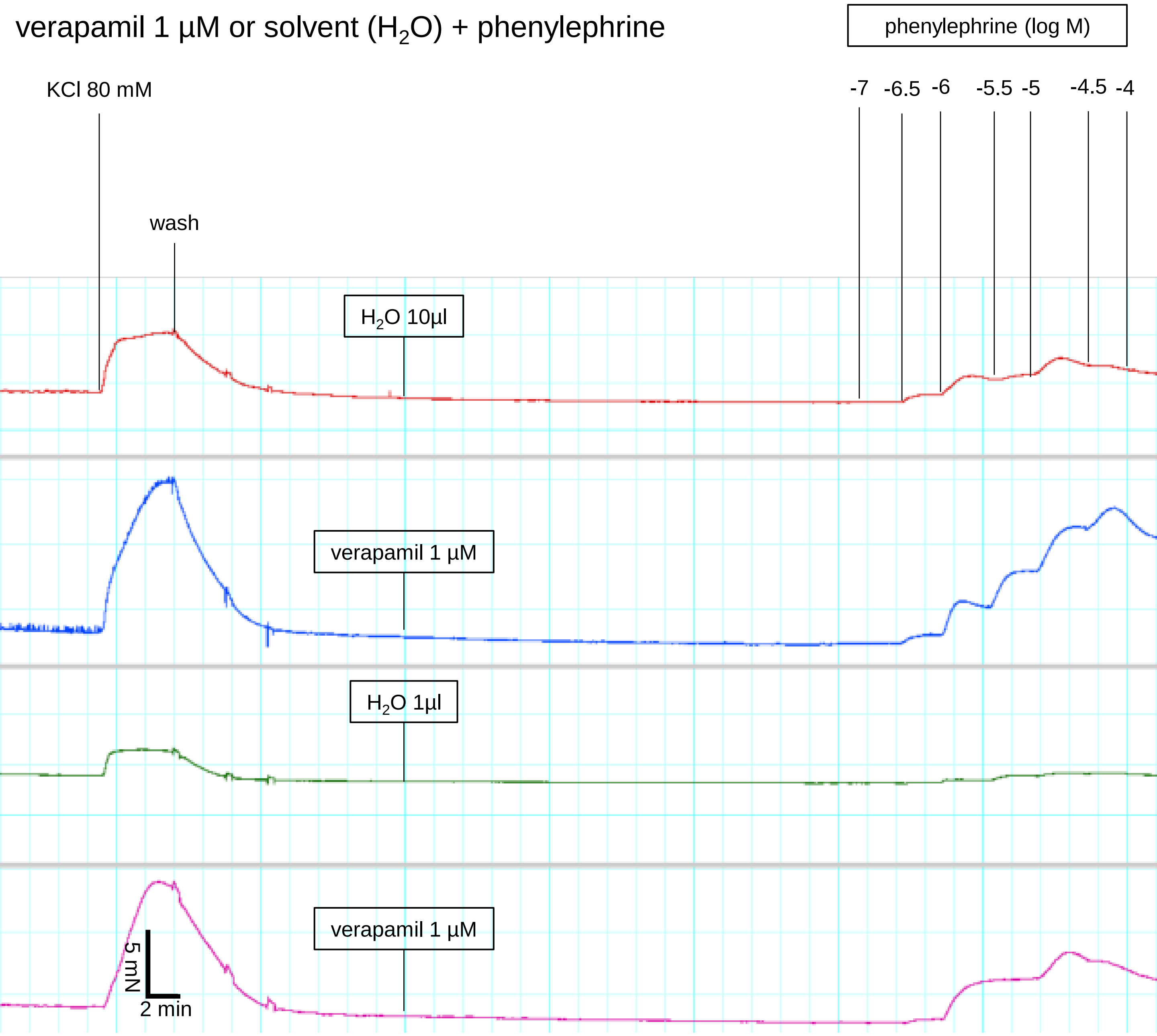

Supplemental Figure 3: Original tracings from an experiment adressing effects of 1 µM verapamil or solvent (control for verapamil) on concentration response curves for phenylephrine. All four curves were recorded with tissues from the same prostate and within the same experiment, which belongs to a series of n=6 independent experiments performed with tissues from n=6 patients (fig. 1c).

Antagonism of prostate α_{1A}-adrenoceptors by verapamil in human prostate smooth muscle contraction
Sheng Hu, Guangyang Liu, Oluwafemi Kale, Wenbin Zhu, Yajie Xu, Patrick Keller, Philipp Weinhold, Alexander Tamalunas, Christian G. Stief, Martin Hennenberg
Journal of Pharmacology and Experimental Therapeutics
JPET-D-25-00098R1

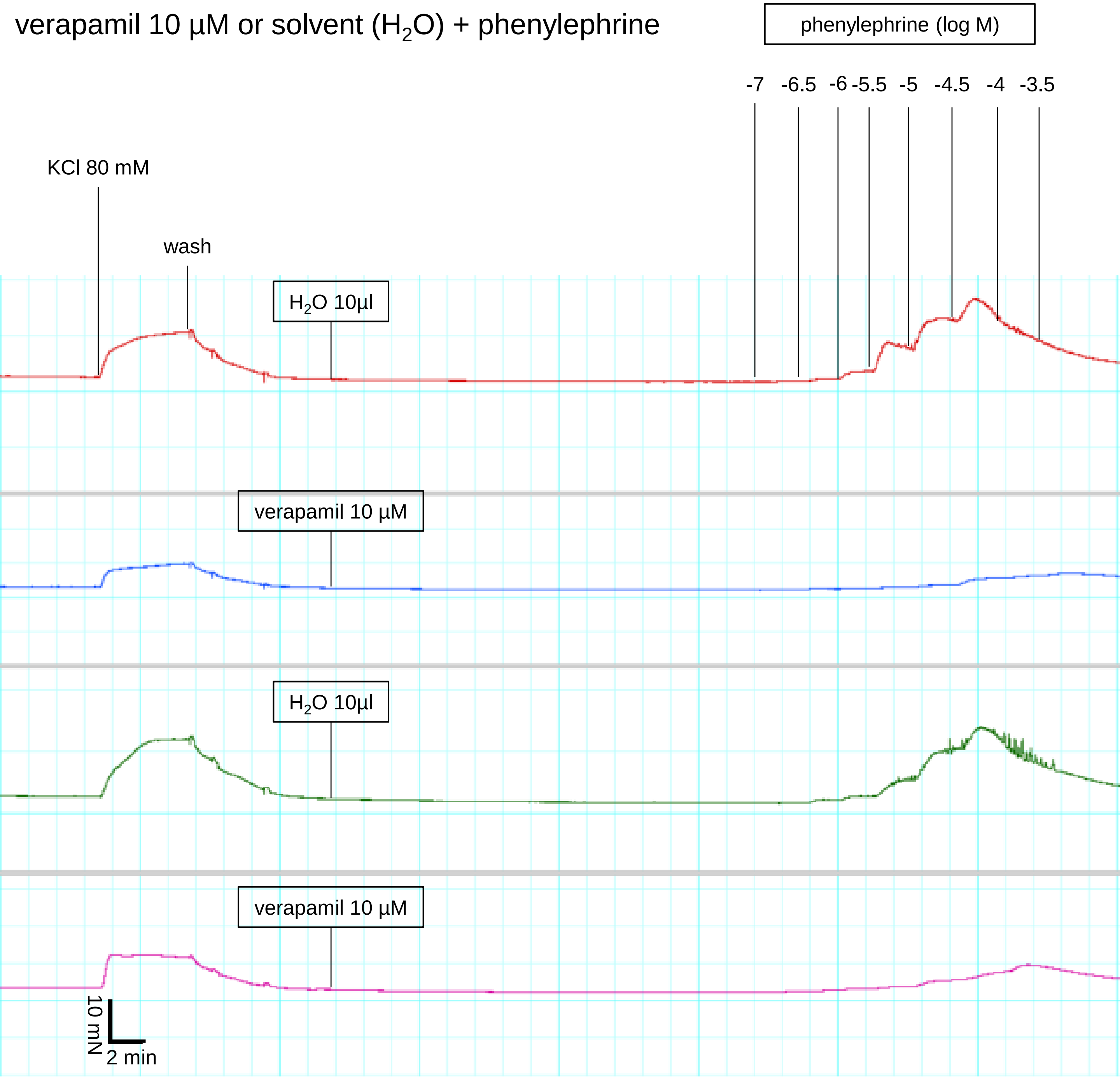

Supplemental Figure 4: Original tracings from an experiment adressing effects of 10 µM verapamil or solvent (control for verapamil) on concentration response curves for phenylephrine. All four curves were recorded with tissues from the same prostate and within the same experiment, which belongs to a series of n=5 independent experiments performed with tissues from n=5 patients (fig. 1d).

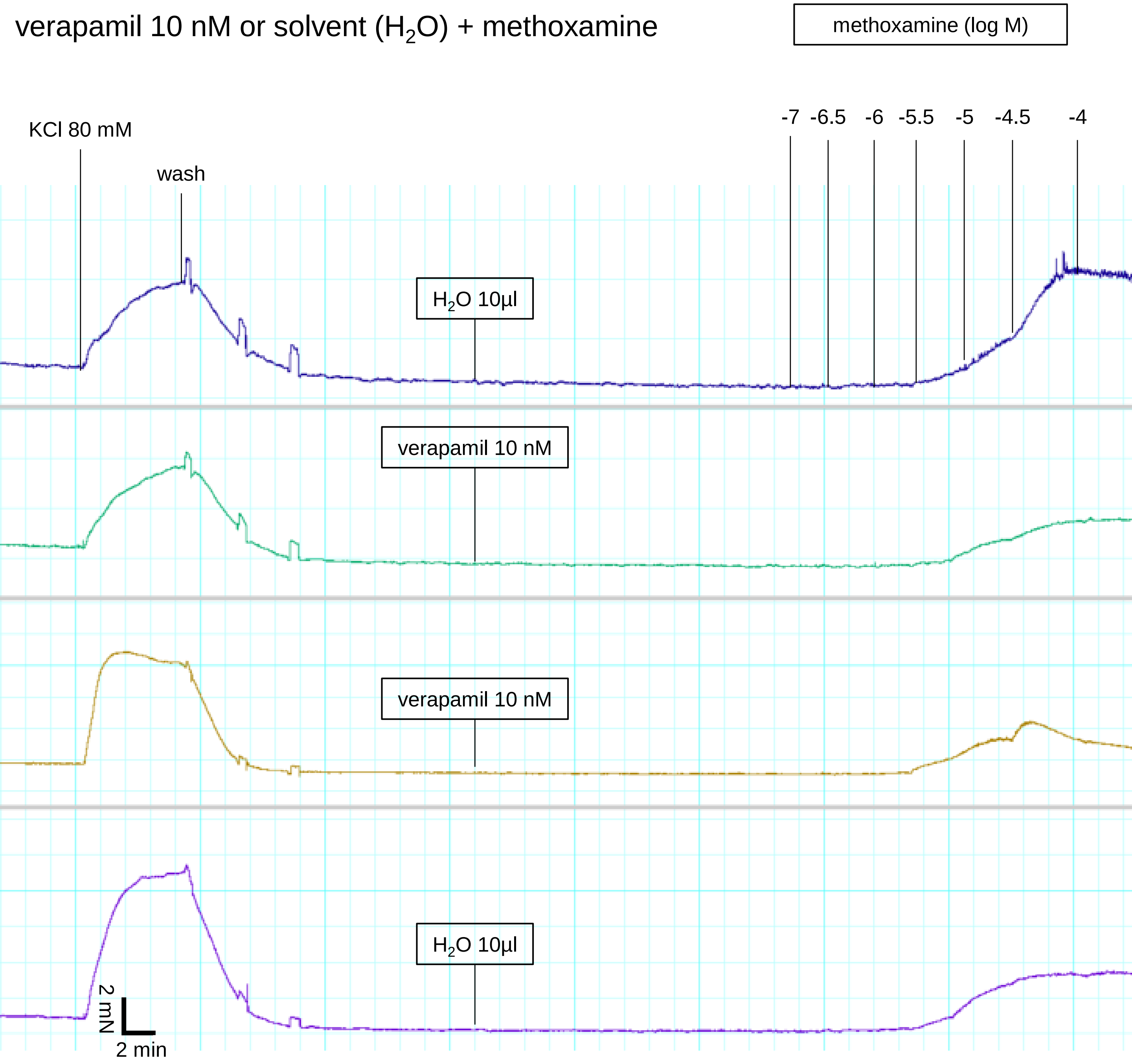

Supplemental Figure 5: Original tracings from an experiment adressing effects of 10 nM verapamil or solvent (control for verapamil) on concentration response curves for methoxamine. All four curves were recorded with tissues from the same prostate and within the same experiment, which belongs to a series of n=5 independent experiments performed with tissues from n=5 patients (fig. 2a).

Antagonism of prostate α_{1A}-adrenoceptors by verapamil in human prostate smooth muscle contraction
Sheng Hu, Guangyang Liu, Oluwafemi Kale, Wenbin Zhu, Yajie Xu, Patrick Keller, Philipp Weinhold, Alexander Tamalunas, Christian G. Stief, Martin Hennenberg
Journal of Pharmacology and Experimental Therapeutics
JPET-D-25-00098R1

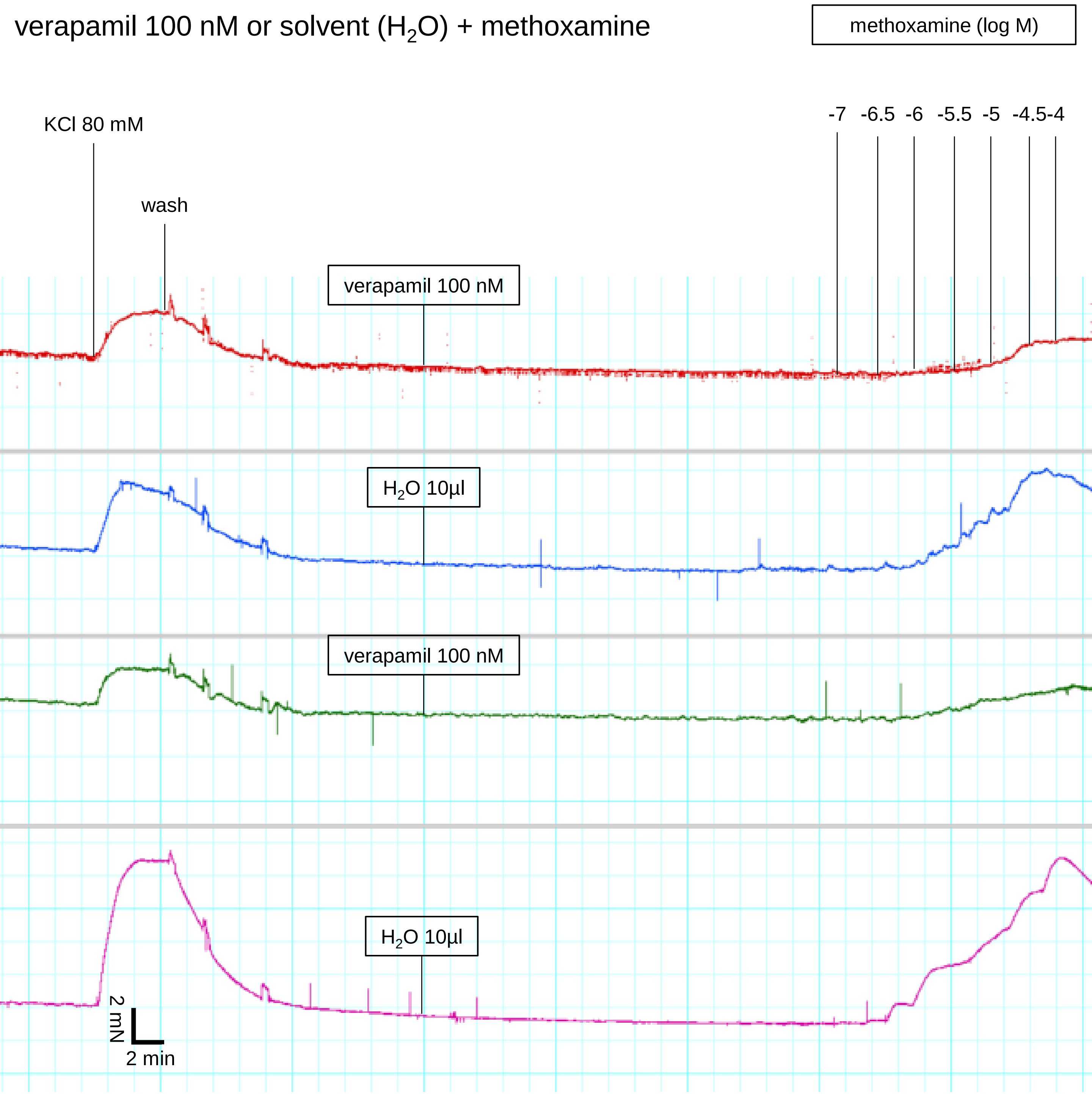

Supplemental Figure 6: Original tracings from an experiment adressing effects of 100 nM verapamil or solvent (control for verapamil) on concentration response curves for methoxamine. All four curves were recorded with tissues from the same prostate and within the same experiment, which belongs to a series of n=7 independent experiments performed with tissues from n=7 patients (fig. 2b).

Antagonism of prostate α_{1A}-adrenoceptors by verapamil in human prostate smooth muscle contraction
Sheng Hu, Guangyang Liu, Oluwafemi Kale, Wenbin Zhu, Yajie Xu, Patrick Keller, Philipp Weinhold, Alexander Tamalunas, Christian G. Stief, Martin Hennenberg
Journal of Pharmacology and Experimental Therapeutics
JPET-D-25-00098R1

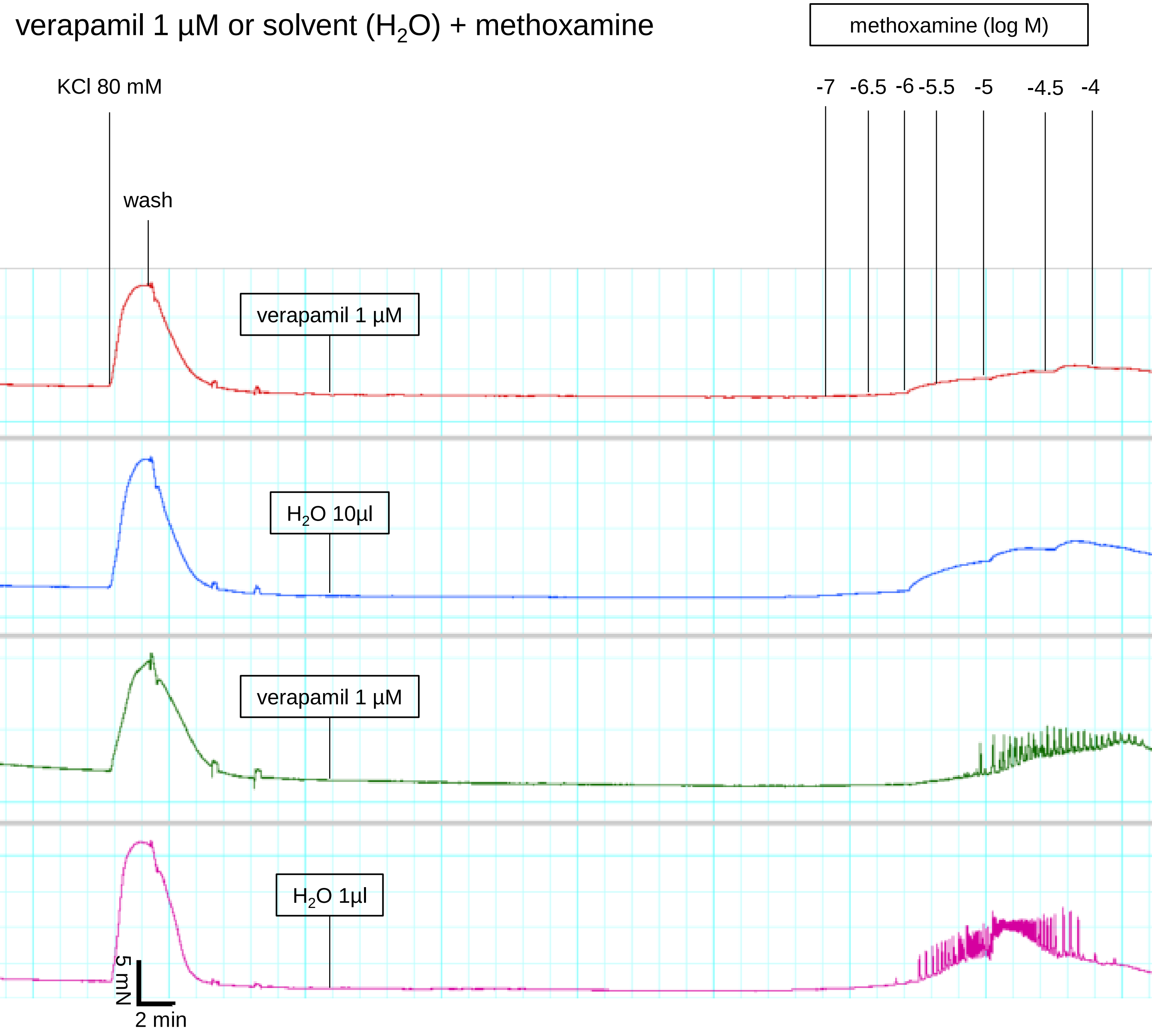

Supplemental Figure 7: Original tracings from an experiment adressing effects of 1 µM verapamil or solvent (control for verapamil) on concentration response curves for methoxamine. All four curves were recorded with tissues from the same prostate and within the same experiment, which belongs to a series of n=5 independent experiments performed with tissues from n=5 patients (fig. 2c).

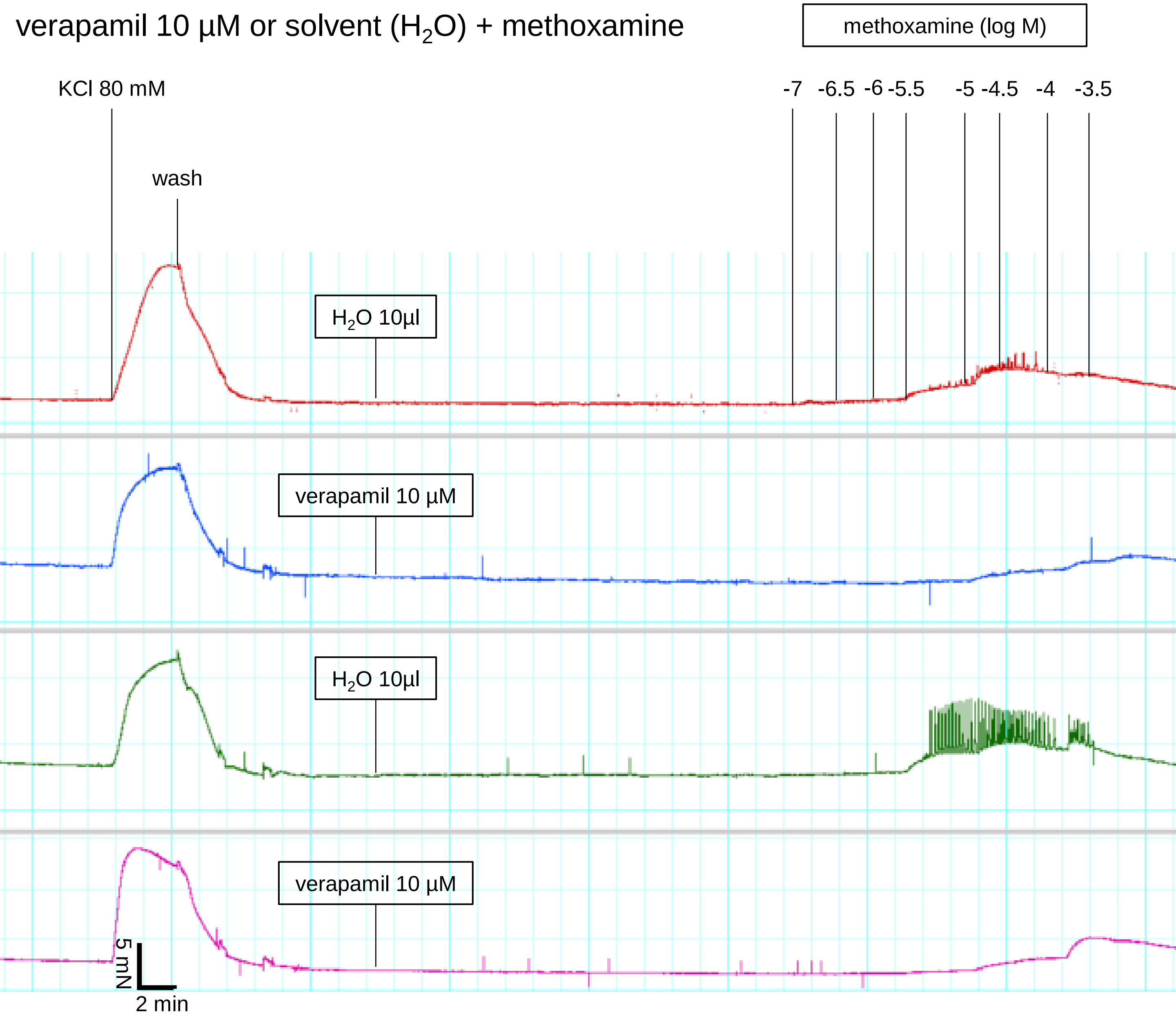

Supplemental Figure 4: Original tracings from an experiment adressing effects of 10 µM verapamil or solvent (control for verapamil) on concentration response curves for methoxamine. All four curves were recorded with tissues from the same prostate and within the same experiment, which belongs to a series of n=5 independent experiments performed with tissues from n=5 patients (fig. 2d).

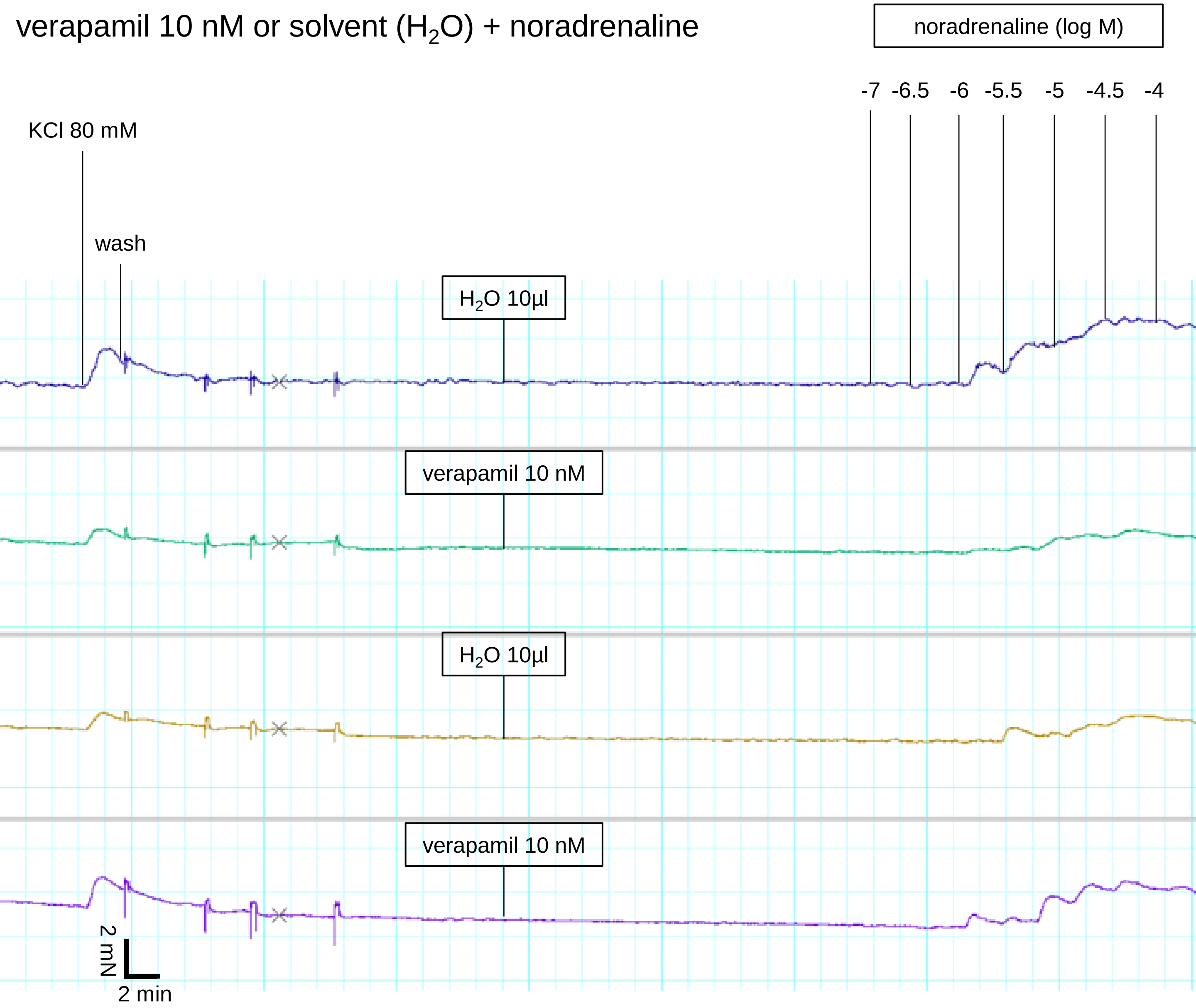

Supplemental Figure 9: Original tracings from an experiment adressing effects of 10 nM verapamil or solvent (control for verapamil) on concentration response curves for noradrenaline. All four curves were recorded with tissues from the same prostate and within the same experiment, which belongs to a series of n=5 independent experiments performed with tissues from n=5 patients (fig. 3a).

Antagonism of prostate α_{1A}-adrenoceptors by verapamil in human prostate smooth muscle contraction
Sheng Hu, Guangyang Liu, Oluwafemi Kale, Wenbin Zhu, Yajie Xu, Patrick Keller, Philipp Weinhold, Alexander Tamalunas, Christian G. Stief, Martin Hennenberg
Journal of Pharmacology and Experimental Therapeutics
JPET-D-25-00098R1

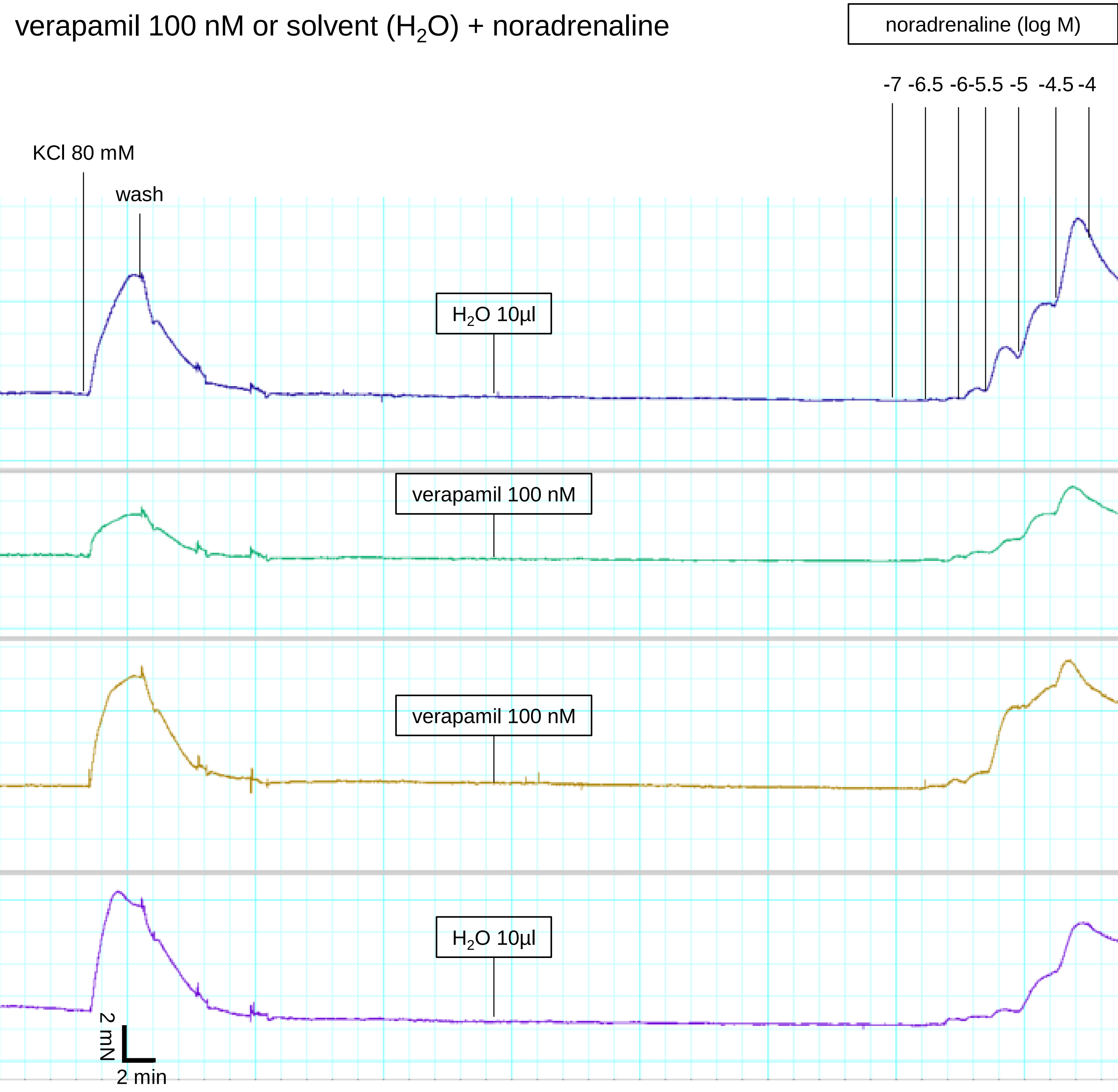

Supplemental Figure 10: Original tracings from an experiment adressing effects of 100 nM verapamil or solvent (control for verapamil) on concentration response curves for noradrenaline. All four curves were recorded with tissues from the same prostate and within the same experiment, which belongs to a series of n=5 independent experiments performed with tissues from n=5 patients (fig. 3b).

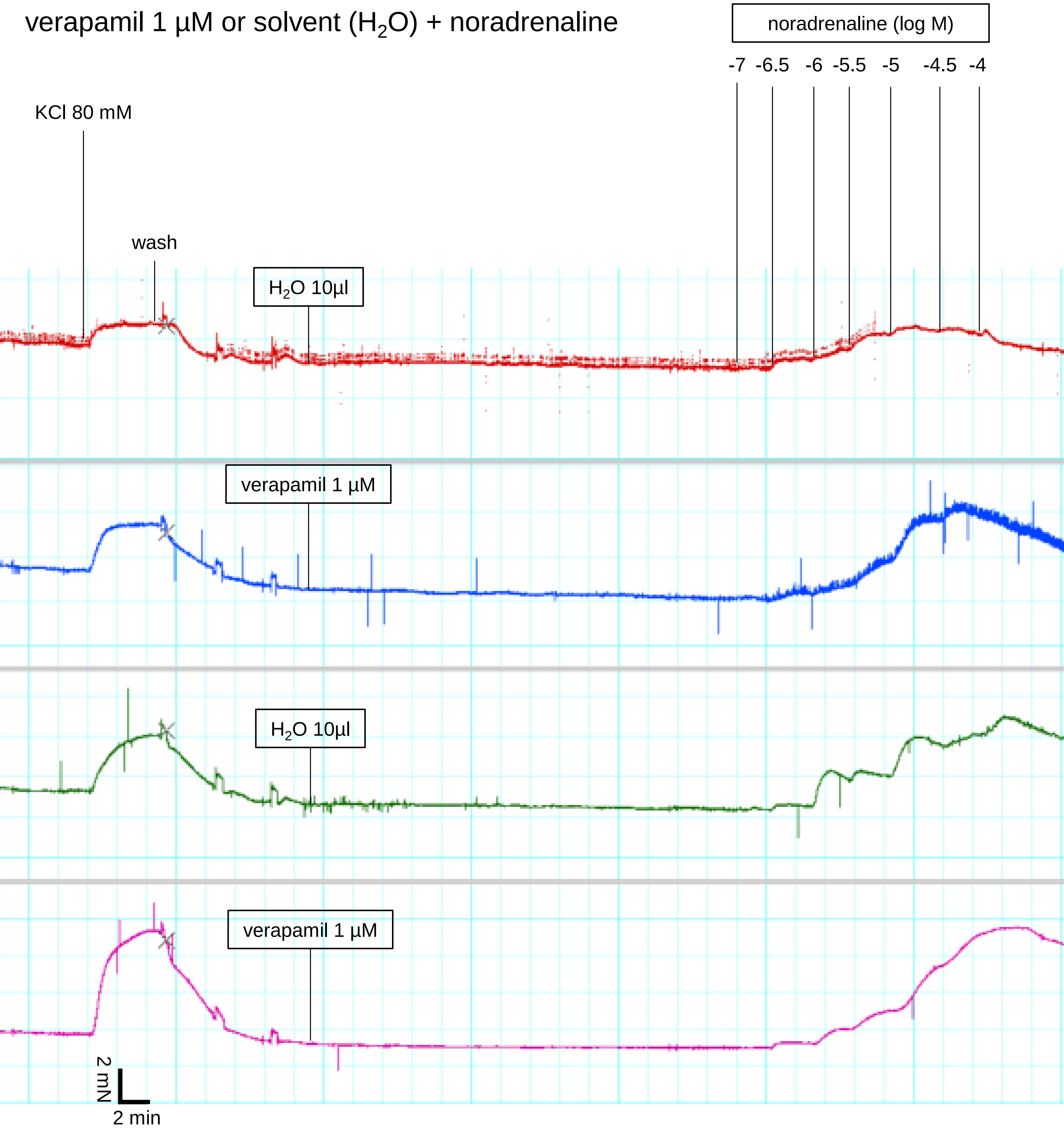

Supplemental Figure 11: Original tracings from an experiment adressing effects of 1 µM verapamil or solvent (control for verapamil) on concentration response curves for noradrenaline. All four curves were recorded with tissues from the same prostate and within the same experiment, which belongs to a series of n=5 independent experiments performed with tissues from n=5 patients (fig. 3c).

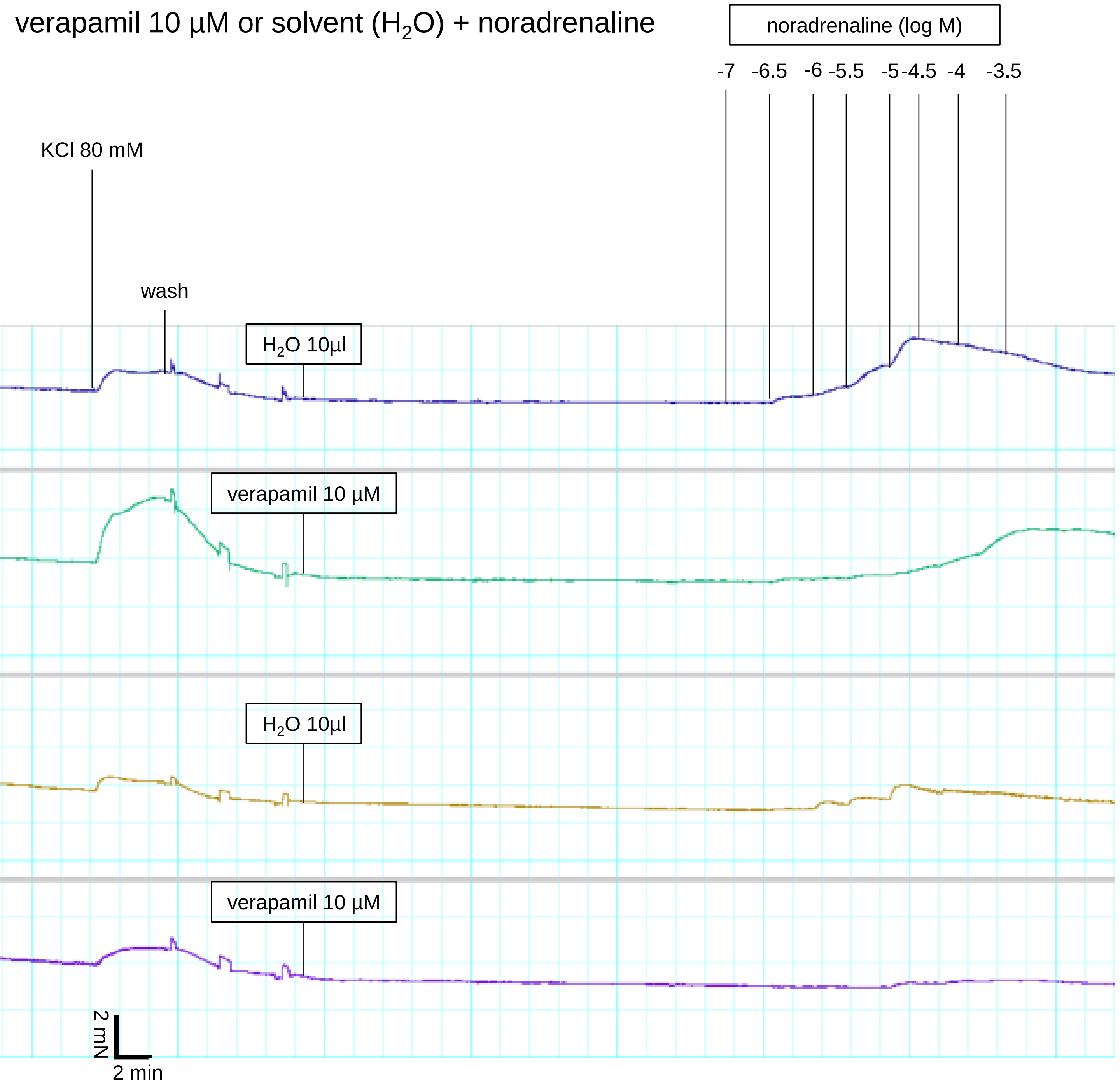

Supplemental Figure 12: Original tracings from an experiment adressing effects of 10 µM verapamil or solvent (control for verapamil) on concentration response curves for noradrenaline. All four curves were recorded with tissues from the same prostate and within the same experiment, which belongs to a series of n=5 independent experiments performed with tissues from n=5 patients (fig. 3d).

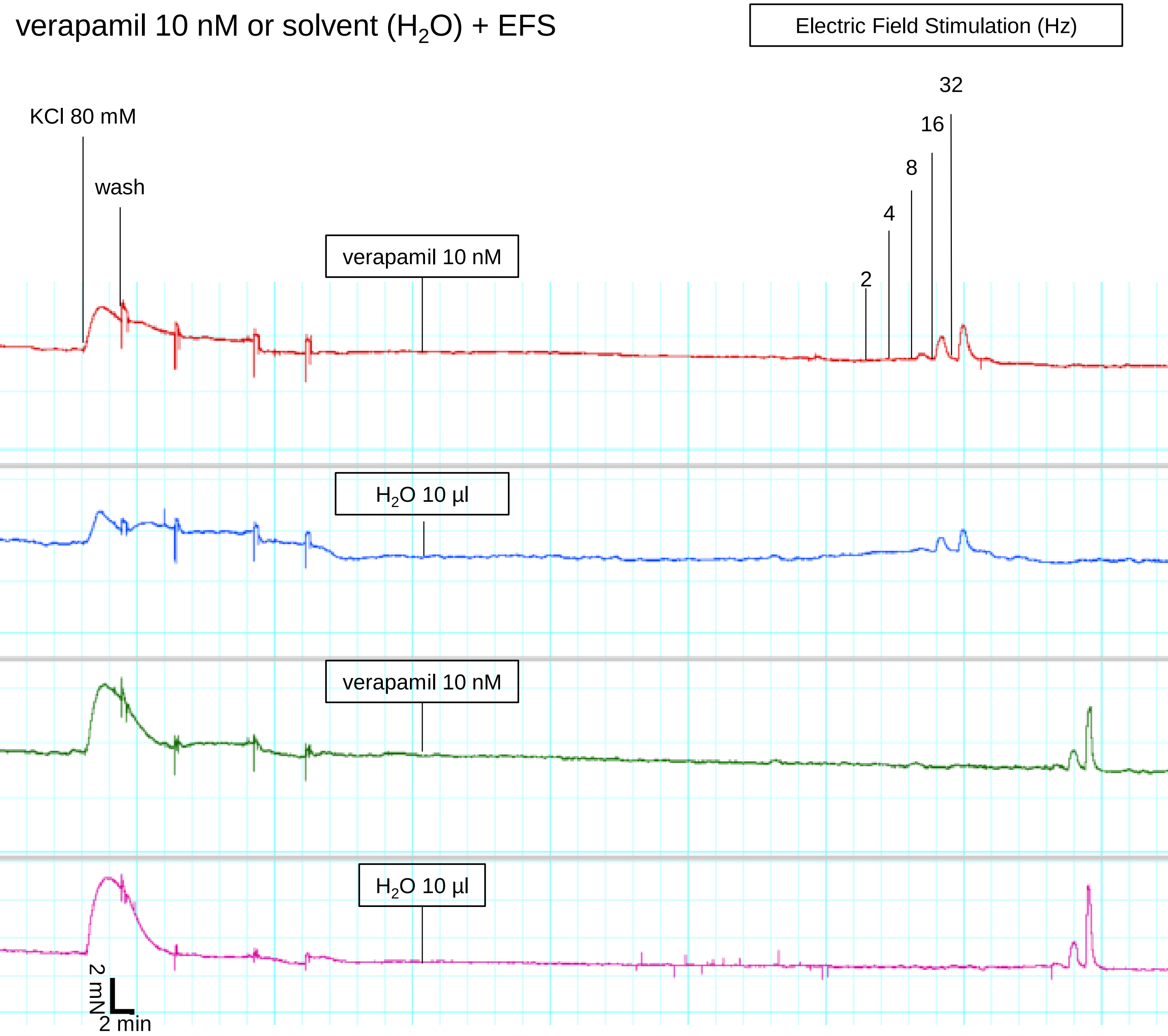

Supplemental Figure 13: Original tracings from an experiment adressing effects of 10 nM verapamil or solvent (control for verapamil) on frequency response curves for electric field stimulation (EFS). All four curves were recorded with tissues from the same prostate and within the same experiment, which belongs to a series of n=5 independent experiments performed with tissues from n=5 patients (fig. 5a).

Antagonism of prostate α$_{1A}$-adrenoceptors by verapamil in human prostate smooth muscle contraction
Sheng Hu, Guangyang Liu, Oluwafemi Kale, Wenbin Zhu, Yajie Xu, Patrick Keller, Philipp Weinhold, Alexander Tamalunas, Christian G. Stief, Martin Hennenberg
Journal of Pharmacology and Experimental Therapeutics
JPET-D-25-00098R1

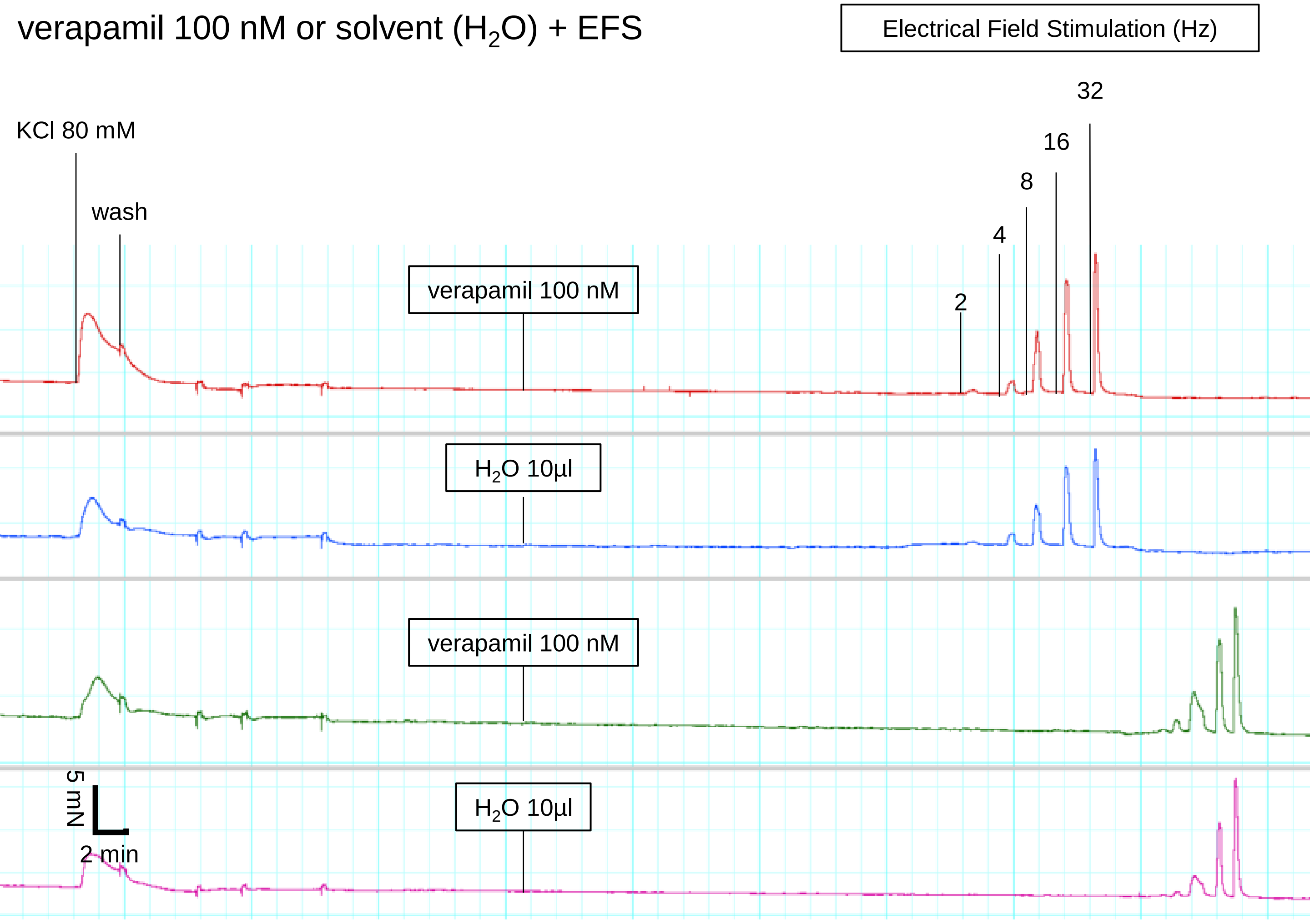

Supplemental Figure 14: Original tracings from an experiment adressing effects of 100 nM verapamil or solvent (control for verapamil) on frequency response curves for electric field stimulation (EFS). All four curves were recorded with tissues from the same prostate and within the same experiment, which belongs to a series of n=7 independent experiments performed with tissues from n=7 patients (fig. 5b).

Antagonism of prostate α_{1A}-adrenoceptors by verapamil in human prostate smooth muscle contraction
Sheng Hu, Guangyang Liu, Oluwafemi Kale, Wenbin Zhu, Yajie Xu, Patrick Keller, Philipp Weinhold, Alexander Tamalunas, Christian G. Stief, Martin Hennenberg
Journal of Pharmacology and Experimental Therapeutics
JPET-D-25-00098R1

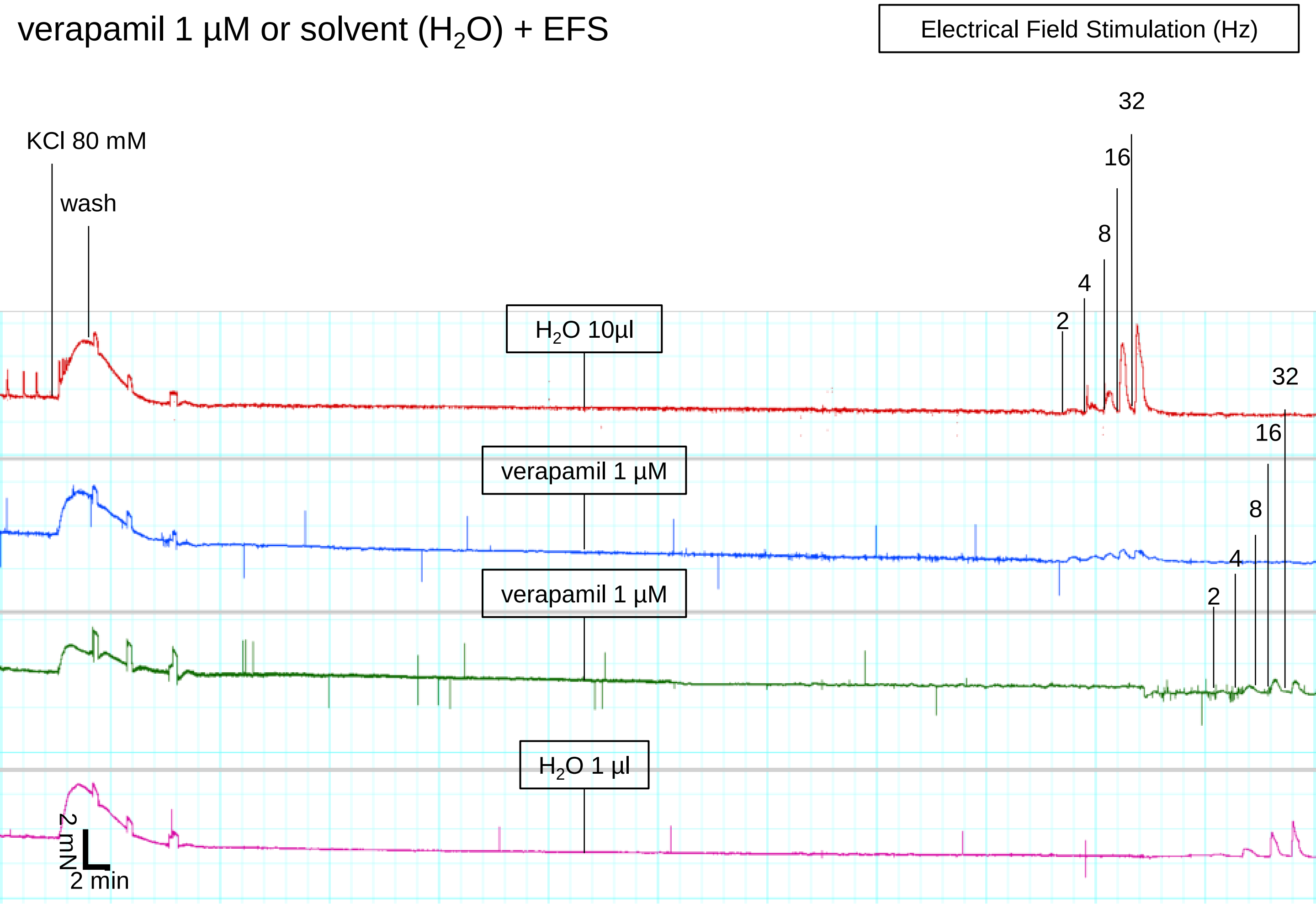

Supplemental Figure 15: Original tracings from an experiment adressing effects of 1 µM verapamil or solvent (control for verapamil) on frequency response curves for electric field stimulation (EFS). All four curves were recorded with tissues from the same prostate and within the same experiment, which belongs to a series of n=5 independent experiments performed with tissues from n=5 patients (fig. 5c).

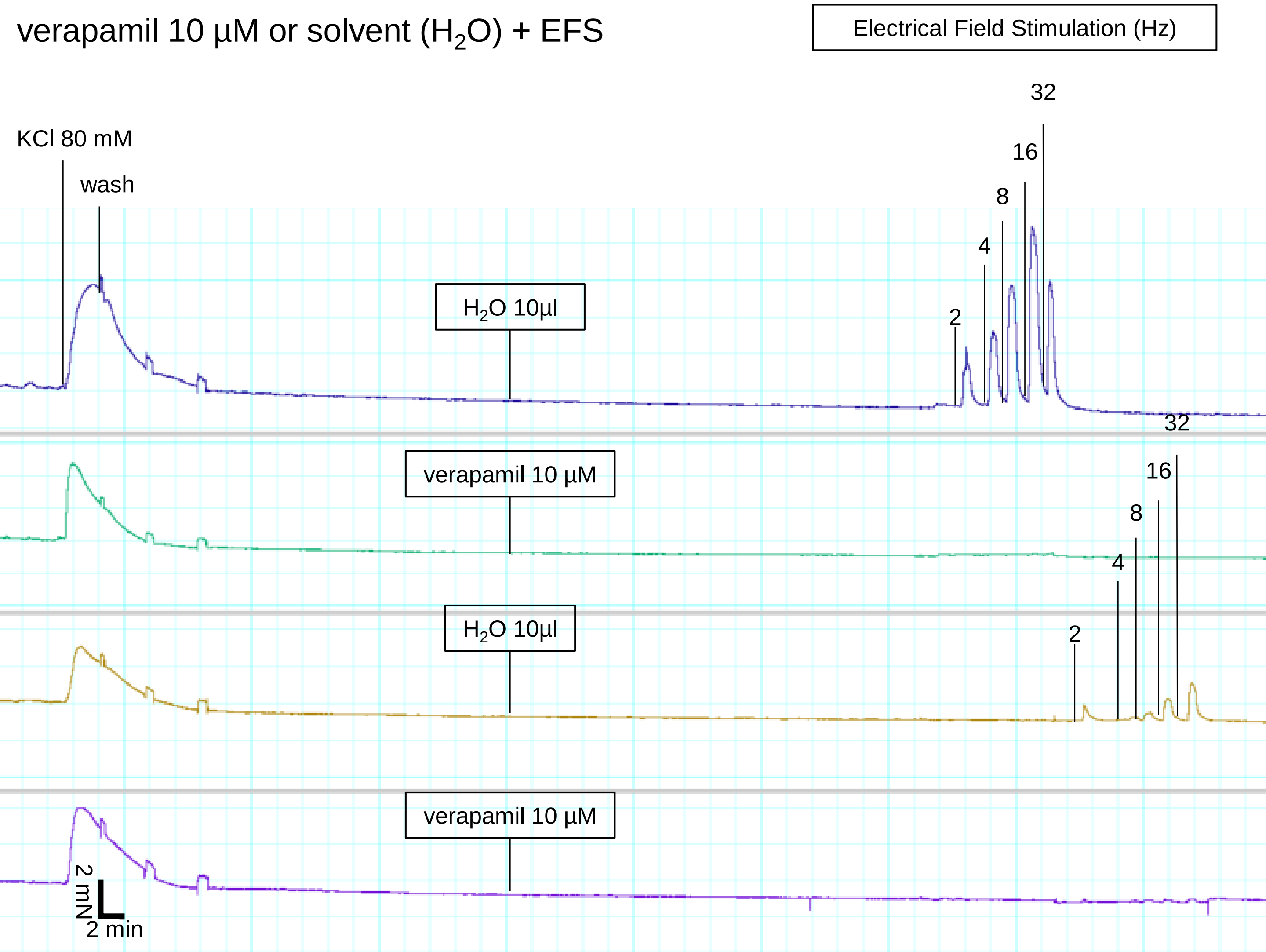

Supplemental Figure 16: Original tracings from an experiment adressing effects of 10 µM verapamil or solvent (control for verapamil) on frequency response curves for electric field stimulation (EFS). All four curves were recorded with tissues from the same prostate and within the same experiment, which belongs to a series of n=5 independent experiments performed with tissues from n=5 patients (fig. 5d).

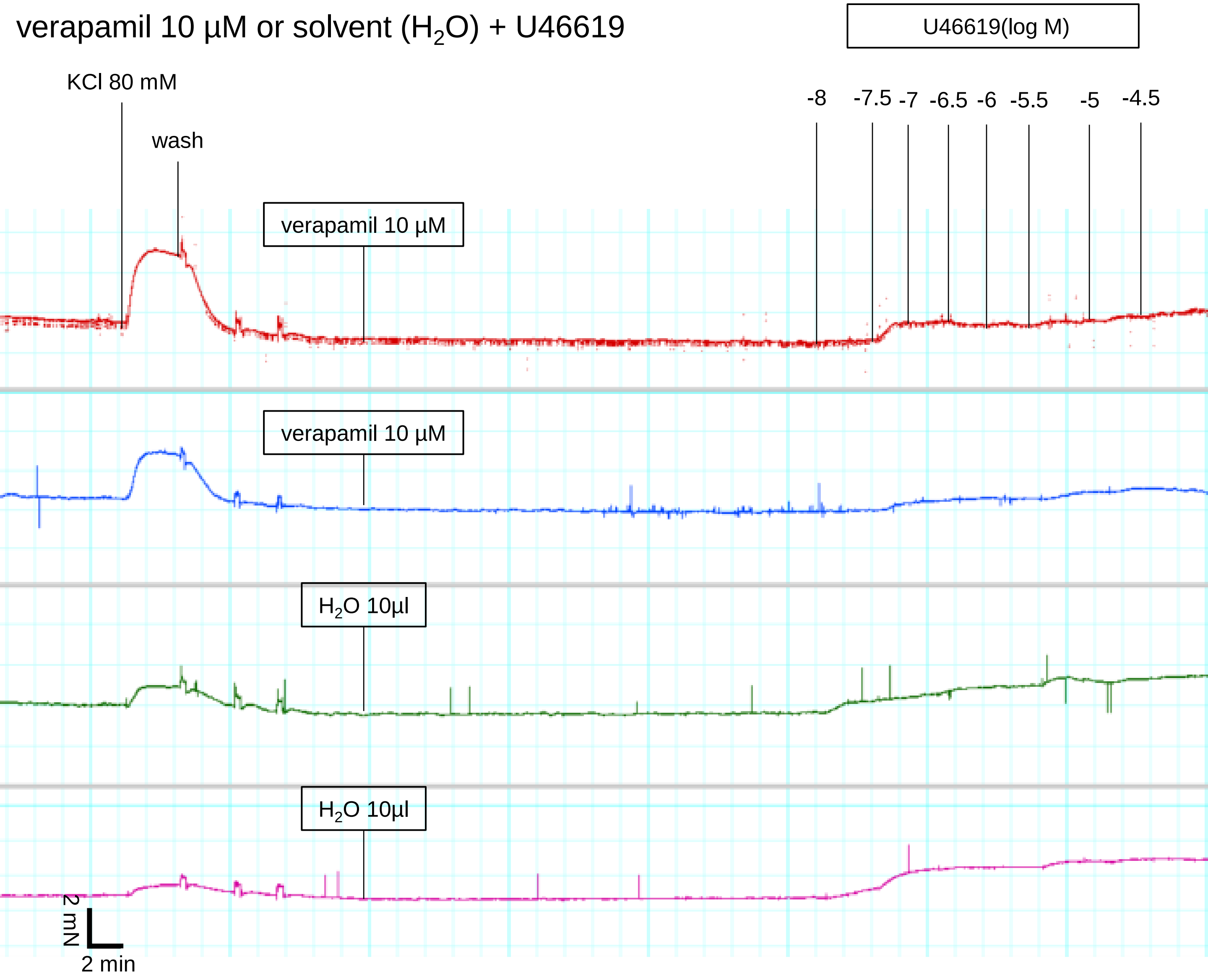

Supplemental Figure 17: Original tracings from an experiment adressing effects of 10 µM verapamil or solvent (control for verapamil) on concentration response curves for U46619. All four curves were recorded with tissues from the same prostate and within the same experiment, which belongs to a series of n=5 independent experiments performed with tissues from n=5 patients (fig. 6a).

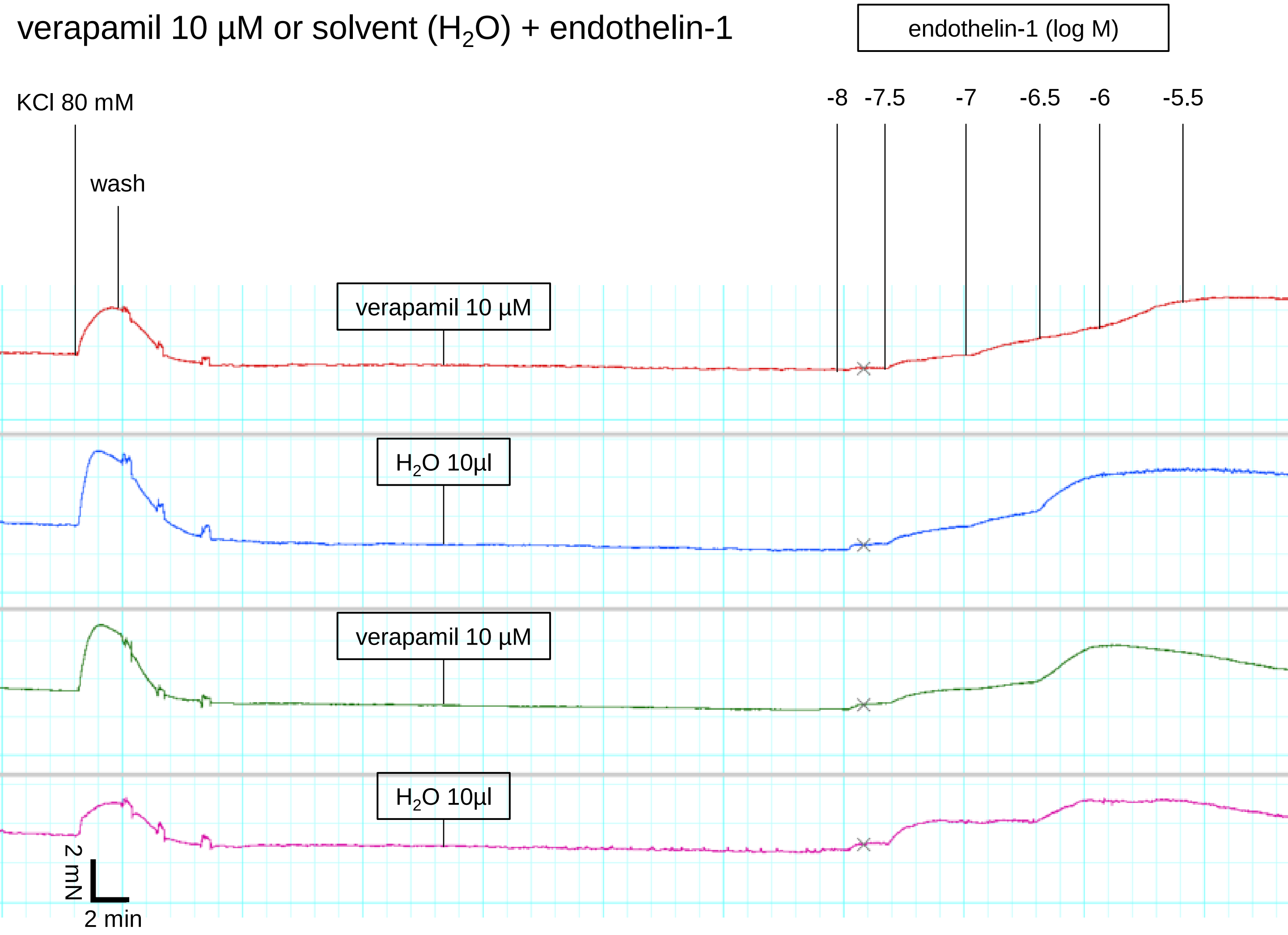

Supplemental Figure 18: Original tracings from an experiment adressing effects of 10 µM verapamil or solvent (control for verapamil) on concentration response curves for endothelin-1. All four curves were recorded with tissues from the same prostate and within the same experiment, which belongs to a series of n=5 independent experiments performed with tissues from n=5 patients (fig. 6b).
